# Inflammatory and nutritional indices as predictors of poor prognosis in pyogenic liver abscesses: a retrospective cohort study

**DOI:** 10.7717/peerj.21852

**Published:** 2026-10-27

**Authors:** Bihong Sun, Zhelong Liu, Shaohua Liu, Tian Yu, Li Liu

**Affiliations:** 1 Department of Endocrinology, Tongji Hospital, Tongji Medical College, Huazhong University of Science and Technology, Wuhan, Hubei, China; 2 Hubei Provincial Clinical Medical Research Center for Endocrinology and Metabolic Diseases, Wuhan, Hubei, China; 3 Branch of National Clinical Research Center for Metabolic Diseases, Wuhan, Hubei, China; 4 Department of Endocrinology, Chongqing Hospital, Union Hospital, Tongji Medical College, Huazhong University of Science and Technology, Wuhan, Hubei, China

**Keywords:** Pyogenic liver abscess, Liver disease, Inflammation-based index, Nutrition-based index, Prognosis, Poor prognosis

## Abstract

**Objective:**

This retrospective study of 458 pyogenic liver abscesses (PLA) patients explored the link between inflammation/nutrition-based indices and poor prognosis in PLA, aiming to enhance the prediction of poor prognosis such as mortality, septic shock, or intensive care unit (ICU) admission.

**Methods:**

Patient data, including demographics, comorbidities, clinical presentations, laboratory results, and treatment outcomes, were extracted from medical records. Multivariable logistic regression and receiver operating characteristic (ROC) curve analyses were performed to identify independent predictors and evaluate their discriminative power. A simplified joint prediction model was developed based on rigorous variable selection criteria, and its performance was assessed using internal validation.

**Results:**

Patients with poor prognosis (*n* = 33) exhibited lower lymphocyte counts, hemoglobin, platelets, and albumin, alongside elevated inflammation markers. Multivariable analysis identified the neutrophil-to-albumin ratio (NAR) (odds ratio (OR) 6.87 per standard deviation (SD), 95% confidence interval (CI) [2.13–22.17], *p* = 0.001), prognostic nutritional index (PNI) (OR 0.99 per SD, 95% CI [0.894–0.997], *p* = 0.038), and controlling nutritional status (CONUT) score (OR 1.37 per SD, 95% CI [1.164–1.602], *p* < 0.001) as independent predictors. ROC analysis showed these indices had good discriminative ability (PNI: area under the curve (AUC) = 0.757, *p* < 0.001; PLR: AUC = 0.704, *p* < 0.001; CONUT: AUC = 0.803, *p* < 0.001; PLT: AUC = 0.740, *p* < 0.001). Notably, a joint prediction model combining platelet count (PLT) and CONUT achieved superior prediction with an AUC of 0.822.

**Conclusion:**

This investigation reveals that easily obtainable inflammatory and nutritional indices (NAR, PNI, CONUT and PLT) serve as reliable predictors of poor prognosis in PLA. A combined model integrating PLT and CONUT demonstrates promising discriminative performance, enabling early risk stratification.

## Introduction

Pyogenic liver abscess (PLA) is a severe infection characterized by hepatic necrosis and systemic inflammation, with mortality rates ranging from 2% to 31% (Sartelli et al., 2017) despite advancements in diagnosis and treatment (Amarantov, Pavlova & Barinova, 2025; Yang et al., 2022). Predicting poor prognosis such as septic shock, ICU admission, and mortality remains difficult due to the heterogeneous clinical course and complex interactions among host immunity, microbial virulence, and metabolic disturbances (Singer et al., 2016). Given these challenges, there is a need to explore novel indicators, such as inflammation and nutrition-based indices, to better understand and predict the prognosis of PLA.

In recent years, inflammation and nutrition-based indices have gained attention for their potential to reflect the interplay between systemic inflammation and nutritional status in various diseases, including cancer and other chronic conditions (Jia et al., 2024; Song et al., 2022). For example, systemic inflammatory indices such as the neutrophil-to-lymphocyte ratio (NLR) and platelet-to-lymphocyte ratio (PLR) have been widely studied for their prognostic value in conditions like liver transplantation and other digestive system disease (Cai et al., 2024; Fang et al., 2020; Niu et al., 2024). Similarly, nutrition-based indices like the prognostic nutritional index (PNI) (Li et al., 2023; Suzuki et al., 2023), controlling nutritional status (CONUT) score (Shi et al., 2023; Spoletini et al., 2021), hemoglobin-albumin-lymphocyte-platelet (HALP) score (Wang et al., 2023; Wang et al., 2024; Xu et al., 2023), neutrophil-to-albumin ratio (NAR) (Bao et al., 2024), and inflammatory burden index (IBI) (Gürbüzer & Ozkaya, 2024; Xie et al., 2023; Yang et al., 2022) have demonstrated prognostic significance in multiple diseases. The HALP score combines hemoglobin, albumin, lymphocyte count, and platelet count to reflect the overall nutritional and inflammatory status. The NAR links neutrophil count, a marker of acute inflammation, with serum albumin, a marker of nutritional status. The IBI quantifies the overall inflammatory burden by combining multiple inflammatory markers. The PNI assesses the overall nutritional and immune status by combining serum albumin and lymphocyte count. The CONUT score evaluates nutritional status by integrating serum albumin, total lymphocyte count, and total cholesterol levels. These indices integrate easily accessible clinical parameters and offer a non-invasive and cost-effective approach to assess disease severity and predict outcomes (Li et al., 2023; Ou et al., 2024; Sugawara et al., 2022). Their potential utility in PLA, however, remains underexplored. Given the established role of inflammation in the progression of liver diseases, it is plausible that these indices could provide valuable insights into the prognosis of PLA.

Therefore, this study aims to address this critical gap by being the first to systematically validate and compare a comprehensive panel of inflammation-nutrition indices (NAR, PNI, CONUT, IBI, HALP, *etc.*) for prognostic prediction in PLA. More importantly, we construct and validate a novel, readily applicable joint prediction model that integrates these indices. This approach moves beyond single-marker analysis and provides a superior, multidimensional tool for early risk stratification, which holds significant promise for guiding timely clinical intervention in PLA patients.

## Methods

### Study design and participants

This retrospective study analyzed a consecutive series of 458 patients with PLA admitted to Tongji Hospital, Tongji Medical College, Huazhong University of Science and Technology, from October 2011 to June 2021. Patients were identified *via* medical records using the search term “pyogenic liver abscess” (ICD-10 code K750). Inclusion criteria were: (1) diagnosis of PLA confirmed by imaging (MRI, CT, or US) showing liver abscess lesions, along with clinical manifestations (fever, chills, liver tenderness), resolution of lesions with antibiotics, or histopathological confirmation (imaging was essential, while other criteria were supportive); (2) age ≥14 years (Xie et al., 2024); and (3) availability of complete laboratory and imaging data. Exclusion criteria followed established research protocols: (1) incomplete clinical data; (2) amebic or parasitic liver abscess (confirmed by serology/stool examination) (Hughes & Petri Jr, 2000); and (3) underlying conditions that may seriously interfere with inflammatory or nutritional status, such as hematologic malignancies, chemotherapy, cirrhosis, or chronic heart failure underlying conditions predisposing to thrombocytopenia (*e.g.*, hematologic malignancies, chemotherapy, cirrhosis, chronic heart failure). PLA management followed the 2017 WSES guidelines for management of intra-abdominal infections (Sartelli et al., 2017). Generally, parenteral antibiotic therapy for liver abscesses lasts 4 to 6 weeks. Follow-up imaging is usually conducted after a certain period of antibiotic treatment. The timing of these imaging studies is adjusted based on the patient’s clinical improvement, therapeutic response, and the physician’s discretion.

### Data collection

Data extracted from the hospital’s electronic medical record system encompassed patient demographics (age and sex), comorbid conditions (such as diabetes mellitus, hypertension, chronic respiratory disease, cardiovascular disease, gastrointestinal/hepatobiliary disorders, and malignancies), clinical presentations (fever, nausea/vomiting, abdominal pain/distension, diarrhea, fatigue), admission vital signs (temperature, heart rate, respiratory rate, blood pressure), initial laboratory results (including complete blood count, liver and renal function tests, inflammatory markers, coagulation profile, lipid levels, random blood glucose, troponin I, NT-proBNP, and other relevant parameters), microbiological culture results, therapeutic interventions, imaging findings, severe complications (septic shock, acute renal/hepatic failure, heart failure, myocardial infarction, pulmonary edema/infection, Acute Respiratory Distress Syndrome (ARDS), pleural effusion) and poor prognosis.

#### Definitions

Poor prognosis was defined as the occurrence of any of the following: death, shock, or ICU admission. Patients were then categorized into poor prognosis group and non-poor prognosis group based on the occurrence of this endpoint. Septic shock was characterized by acute circulatory failure with lactate >2 mmol/L, persistent hypotension unexplained by other causes, even after adequate fluid resuscitation and vasopressor requirement (Singer et al., 2016). Because our cohort spanned the years 2011 to 2021, and the Sepsis-3 definitions were published in 2016, all patients (including those admitted before 2016) were retrospectively re-classified according to the Sepsis-3 criteria at the time of data collection in 2022, in order to ensure a uniform definition across the entire study period. Acute renal failure was identified by serum creatinine levels exceeding 176 µmol/L or an absolute increase of more than 44 µmol/L (Englberger et al., 2011). Severe acute liver injury was defined as alanine aminotransferase (ALT) or aspartate aminotransferase (AST) levels exceeding 5 times the upper limit of normal (ULN), in accordance with previously published definitions (Mueller et al., 2019). Heart failure was defined in line with the guidelines from the Heart Failure Association of the European Society of Cardiology (McDonagh et al., 2024). Myocardial infarction was determined by serum hypersensitive cardiac troponin I (hs-cTnI) levels above 34 pg/ml (Thygesen et al., 2019). Pulmonary edema was primarily diagnosed based on pulmonary imaging findings. ARDS was defined following The Berlin Definition of Acute Respiratory Distress Syndrome (Ranieri et al., 2012).

#### Statistical analysis

Continuous variables were expressed as mean ± standard deviation or median (interquartile range), while categorical variables were reported as frequencies. Baseline characteristics were compared between adverse-outcome and non-adverse-outcome groups using Student’s *t*-test, Mann–Whitney U-test, or chi-square test. Multivariable logistic regression adjusted for confounders (age, sex, comorbidities) was performed to identify independent predictors. To develop a joint prediction model, candidate variables were selected based on demonstrating statistical significance in both univariate and multivariable logistic regression analyses, as well as achieving an individual AUC greater than 0.7. Bootstrap validation with 1,000 resamples was performed to evaluate the stability of the regression coefficients. The optimal cut-off values for each index were determined using the Youden index (maximum sensitivity + specificity −1) from ROC curve analysis. Receiver operating characteristic (ROC) curves will evaluate the discriminative power of indices for poor prognosis. Statistical significance was set at *P*  < 0.05. Analyses were performed using SPSS 27.0 (IBM Corp., Armonk, NY, USA) and R studio 4.5.2.

#### Ethical considerations

The study received approval from the ethics committee of Tongji Hospital, Huazhong University of Science and Technology, and was conducted in accordance with the Declaration of Helsinki (TJ-IRB20221240). Given its retrospective nature, the study was exempt from informed consent because of its retrospective design.

## Results

### Baseline characteristics and clinical profiles

The study included 458 PLA patients, stratified into poor prognosis (*n* = 33) and non-poor prognosis (*n* = 425) groups. No significant differences were observed in age (52.64 ± 13.46 *vs.* 52.98 ± 13.42 years, *p* = 0.943) or sex distribution (57.6% *vs.* 72.5% male, *p* = 0.068). However, the poor prognosis group exhibited higher frequencies of dyspnea (18.2% *vs.* 0.5%, *p* < 0.001), palpitations (18.2% *vs.* 4.9%, *p* = 0.002), and consciousness disturbance (6.1% *vs.* 1.2%, *p* = 0.028). Comorbidities such as cardiovascular disease were more prevalent in the poor prognosis group (9.1% *vs.* 2.4%, *p* = 0.025). Vital signs at admission revealed lower systolic blood pressure (112 ± 24.76 *vs.* 121 ± 17.87 mmHg, *p* = 0.019) and diastolic blood pressure (69 ± 15.03 *vs.* 76 ± 11.25 mmHg, *p* = 0.024), along with higher heart rates (median 96 *vs.* 89 bpm, *p* = 0.014) in the poor prognosis group. Abscess characteristics (size ≥ 5 cm, gas presence, multiloculation) did not differ significantly (all *p* > 0.05, Table 1).

**Table 1:** Demographic and baseline characteristics of patients between poor prognosis group and non- poor prognosis group with pyogenic liver abscess. Data are presented as mean ± SD or median (interquartile range) for continuous variables and n (%) for categorical variables. *P*-values comparing poor prognosis and non-poor prognosis groups were obtained using Student’s t-test, Mann–Whitney U-test, *χ*^2^ test, or Fisher’s exact test. Poor prognosis included either death, shock, or admission to the ICU. Bold values indicate statistical significance (*P* < 0.05).

Characteristics	poor prognosis (*n* = 33)	Non-poor prognosis (*n* = 425)	*P* value
Age, mean ± SD	52.64 ± 13.46	52.98 ± 13.42	0.943
Sex, n (%)			
Men	19 (57.58)	308 (72.47)	0.068
Women	14 (42.42)	117 (27.53)	0.068
**Clinical symptoms/signs**			
Fever, n (%)	12 (36.36)	107 (25.18)	0.158
Nausea and vomiting, n (%)	6 (18.18)	77 (18.12)	0.993
Abdominal distension or pain, n (%)	12 (36.36)	218 (51.29)	0.098
Diarrhea, n (%)	5 (15.15)	31 (7.29)	0.106
Fatigue and muscle pain, n (%)	18 (54.55)	160 (37.65)	0.055
Chest pain, n (%)	1 (3.03)	17 (4.00)	0.782
Palpitation, n (%)	6 (18.18)	21 (4.94)	**0**.**002**
Cough and sputum, n (%)	6 (18.18)	37 (8.71)	0.072
Dizziness or headache, n (%)	5 (15.15)	44 (10.35)	0.390
Dyspnea, n (%)	6 (18.18)	2 (0.47)	**<0**.**001**
Disturbance of consciousness, n (%)	2 (6.06)	5 (1.18)	**0**.**028**
**Co-morbidity**			
Diabetes mellitus, n (%)	10 (30.30)	91 (21.41)	0.235
Hypertension, n (%)	5 (15.15)	73 (17.18)	0.766
Cardiovascular disease, n (%)	3 (9.09)	10 (2.35)	**0**.**025**
Chronic respiratory disease, n (%)	3 (9.09)	19 (4.47)	0.233
Urologic diseases, n (%)	0 (0.00)	8 (1.88)	0.425
Malignancy, n (%)	2 (6.06)	20 (4.71)	0.726
Liver and gallbladder stones, n (%)	7 (21.21)	93 (21.88)	0.928
Viral hepatitis, n (%)	5 (15.15)	49 (11.53)	0.534
Fatty liver disease, n (%)	4 (12.12)	45 (10.59)	0.784
**Vital sign at admission**			
Temperature, °C	36.7 (36.4–37.5)	36.7 (36.4–37.2)	0.891
SBP, (mmHg) mean ± SD	112 ± 24.76	121 ± 17.87	**0**.**019**
DBP, (mmHg) mean ± SD	69 ± 15.03	76 ± 11.25	**0**.**024**
Heart rates, max/min	96 (68–122)	89 (60–138)	**0**.**014**
Respiratory rates, max/min	22 (12–39)	20 (10–32)	0.111
The size of abscess (≥5 cm), n(%)	1 (3.03)	48 (11.29)	0.238
Presence of gas, n(%)	4 (12.12)	35 (8.24)	0.511
Multiloculation, n(%)	7 (21.21)	77 (18.12)	0.716

**Notes.**

SBP systolic blood pressure DBP diastolic blood pressure

### Baseline laboratory parameters of patients with PLA

The poor prognosis group demonstrated pronounced hematologic and biochemical derangements: lower lymphocyte counts (0.8 *vs.* 1.3 ×10^9^/L, *p* = 0.005), hemoglobin (103 *vs.* 116 g/L, *p* < 0.001), and platelets (74 *vs.* 238 ×10^9^/L, *p* < 0.001), alongside elevated total bilirubin (16.7 *vs.* 11.7 µmol/L, *p* = 0.034), direct bilirubin (9.1 *vs.* 5.3 µmol/L, *p* = 0.001), and AST (35 *vs.* 25 U/L, *p* = 0.025). Markers of systemic inflammation (CRP, PCT) and cardiac stress (NT-proBNP: 1,179 *vs.* 474 pg/mL, *p* = 0.009) were also significantly elevated. Incidentally, the following laboratory parameters showed no statistically significant differences between the adverse and non-poor prognosis groups (*p* ≥ 0.05): white blood cell count, neutrophil count, CRP, PCT, ALT, ALP, γ-GT, random blood glucose, creatinine, and cTnI (Table 2).

**Table 2:** Laboratory indices between poor prognosis group and non- poor prognosis group with pyogenic liver abscess. Data are presented as median (interquartile range) for continuous variables. *P*-values comparing thrombocytopenia and non-thrombocytopenia groups were obtained using the Mann–Whitney U-test. Poor prognosis included either death, shock, or admission to the ICU. Bold values indicate statistical significance (*P* < 0.05).

Laboratory data	Normal range	poor prognosis (*n* = 33)	Non-poor prognosis (*n* = 425)	*P* value
White blood cell,109/L	3.5–9.5	10.9 (7.2, 18.6)	10.1 (7.0, 13.5)	0.616
Neutrophil count,109/L	1.8–6.3	8.8 (6.1, 15.5)	7.8 (4.8, 11.3)	0.256
Lymphocyte count,109L	1.10–3.2	0.8 (0.6, 1.4)	1.3 (0.9, 1.7)	**0**.**005**
Hemoglobin, g/L	115–150	103 (81, 111)	116 (102.5, 129.5)	**<0**.**001**
Platelets median, 109/L	125–350	74 (42, 250)	238 (149, 327)	**<0**.**001**
CRP, mg/L	<1	140 (87, 198)	114.2 (55.3, 187.3)	0.639
PCT, ng/ml	<0.05	3.0 (1.0, 11.3)	0.8 (0.2, 6.8075)	0.082
Total bilirubin median, μ mol/L	≤21	16.7 (8.5, 35.6)	11.7 (7.9, 17.6)	**0**.**034**
Direct bilirubin, μ mol/L	≤8	9.1 (3.7, 25.1)	5.3 (3.0, 8.7)	**0**.**001**
ALT, U/L	<33	37 (18,87)	31 (18, 54)	0.461
AST, U/L	<32	35 (20, 79)	25 (18, 43)	**0**.**025**
ALP, U/L	135–214	154 (115, 279)	138 (98, 207)	0.153
γ-GT, U/L	6–42	124 (54, 154)	115 (63, 196)	0.418
Albumin, g/L	35–52	25.9 ± 1.31	32.1 ± 0.3	**<0**.**001**
TC, μ mol/L	<5.8	2.1 (1.8, 2.8)	3.2 (2.5, 3.8)	**<0**.**001**
TG, μmol/L	<1.7	1.3 (1.0, 1.8)	1.2 (0.8, 1.9)	0.671
PT, s	11.5–14.5	15.8 (14.8, 18.0)	14.6 (13.9, 15.5)	**<0**.**001**
APTT, s	29–42	43.5 (39.1, 46.2)	41.0 (37.0, 44.8)	0.215
Random blood glucose, μ mol/L	≤11.1	7.1 (5.6, 10.9)	7.1 (5.5, 10.7)	0.825
Creatinine median, μ mol/L	59–104	70 (50, 105)	67 (54, 83)	0.573
NT-proBNP, pg/ml	<62.9	1,179 (633, 5,133)	474 (174, 1,948)	**0**.**009**
cTnI, pg/mL	≤34.2	2.1 (0.0, 14.2)	0.3 (0.0, 6.2)	0.282

**Notes.**

CRP C-reactive protein PCT procalcitonin ALT alanine aminotransferase AST aspartate aminotransferase ALP alkaline phosphatase γ-GT γ- glutamate transpeptidase TC total cholesterol TG triglyceride PT prothrombin time APTT activated partial thromboplastin time BUN blood urea nitrogen NT-proBNP N-terminal probrain natriuretic peptide cTnI cardiac troponin I

### Inflammation/nutrition-based indicators of patients between poor prognosis group and non-poor prognosis group with PLA

All evaluated indices except HALP (*p* = 0.214) significant associations with poor prognosis (Table 3). The poor prognosis group had higher NLR (11.10 *vs.* 5.95, *p* = 0.001), NAR (0.36 *vs.* 0.25, *p* = 0.001), CONUT (9.20 ± 2.63 *vs.* 5.67 ± 3.04, *p* < 0.001), and IBI (2,182.11 *vs.* 794.23, *p* = 0.003), but lower PLR (91.70 *vs.* 185.61, *p* < 0.001) and PNI (28.93 *vs.* 38.95, *p* < 0.001).

**Table 3:** Characteristics of inflammation/nutrition-based indicators of patients between poor prognosis group and non- poor prognosis group with PLA. Poor prognosis included either death, shock, or admission to the ICU. Bold values indicate statistical significance (*P* < 0.05).

Indicators	Total (*n* = 458)	poor prognosis (*n* = 33)	Non-poor prognosis (*n* = 425)	*P* value
HALP median (IQR)	20 (13–35)	25 (12–53)	20 (13–33)	0.214
NLR median (IQR)	6.15 (3.53–11.88)	11.10 (5.37–18.93)	5.95 (3.49–11.35)	**0**.**001**
PLR median (IQR)	178.55 (111.92–264.01)	91.70 (46.78–195.40)	185.61 (118.46–269.19)	**<0**.**001**
NAR median (IQR)	0.26 (0.15–0.39)	0.36 (0.25–0.70)	0.25 (0.14–0.38)	**0**.**001**
PNI median (IQR)	38.50 (32.73–72.93)	28.93 (24.15–36.95)	38.95 (33.38–44.20)	**<0**.**001**
CONUT (IQR)	6 (4–8)	10 (7–12)	6 (3–8)	**<0**.**001**
IBI median (IQR)	895.86 (194.62–2,163.03)	2,182.11 (1,151.20–2,971.85)	794.23 (184.93–1,998.14)	**0**.**003**

**Notes.**

HALP Hemoglobin, albumin, lymphocyte and platelet scores NLR neutrophil-to-lymphocyte ratio PLR platelet-to-lymphocyte ratio NAR Neutrophil-albumin ratio PNI prognostic nutritional index CONUT score control ling nutritional status score IBI Inflammation burden index

### Treatment and clinical outcomes of patients between poor prognosis group and non-poor prognosis group with PLA

As shown in Table 4, albumin infusion was more frequent in the poor prognosis group (78.8% *vs.* 41.7%, *p* < 0.001). Treatment modalities (antibiotics alone, percutaneous drainage, or surgery) did not differ (all *p* > 0.05). Poor prognosis were associated with higher rates of complications: acute renal injury (15.2% *vs.* 5.4%, *p* = 0.039), heart failure (33.3% *vs.* 4.5%, *p* < 0.001), ARDS (24.2% *vs.* 0.5%, *p* < 0.001), severe acute liver injury (18.18% *vs.* 2.82%, *p* < 0.001) and mortality (45.5% *vs.* 0%, *p* < 0.001) .

**Table 4:** Treatment and clinical outcomes between poor prognosis group and non- poor prognosis group with PLA. Data are presented as n (%). *P*-values comparing poor prognosis group and non-poor prognosis group were obtained using chi-square test or Fisher’s exact test. Antibiotics alone: treatment with antibiotics only; Antibiotics plus percutaneous drainage: treatment with antibiotics combined with percutaneous drainage; Antibiotics plus surgical: treatment with antibiotics combined with surgical intervention. Poor prognosis included either death, shock, or admission to the ICU. Bold values indicate statistical significance (*P* < 0.05).

**Treatments**	Total (*n* = 458)	poor prognosis (*n* = 33)		Non-poor prognosis (*n* = 425)		*P* value
Antibiotics alone	128 (28.00)		9 (27.27)		119 (28.00)	0.929
Antibiotics plus percutaneous drainage	278 (60.7)		19 (57.58)		259 (60.94)	0.703
Antibiotics plus surgical	33 (7.2)		3 (9.09)		30 (7.06)	0.722
Albumin infusion	203 (44.3)		26 (78.79)		177 (41.65)	**<0**.**001**
Antiviral drug	29 (6.3)		4 (12.12)		25 (5.88)	0.319
**Serious complications**				
Acute renal injury	28 (6.2)		5 (15.15)		23 (5.41)	**0**.**039**
Severe acute liver injury	18 (3.93)		6 (18.18)		12 (2.82)	**<0**.**001**
Acute hepatic injury	212 (46.3)		22 (66.67)		190 (44.71)	**0**.**015**
Heart failure	30 (6.6)		11 (33.33)		19 (4.47)	**<0**.**001**
Myocardial infarction	13 (2.8)		6 (18.18)		7 (1.65)	**<0**.**001**
Pulmonary edema	12 (2.6)		9 (27.27)		3 (0.71)	**<0**.**001**
ARDS	10 (2.2)		8 (24.24)		2 (0.47)	**<0**.**001**
Pleural effusion	187 (40.8)		23 (69.70)		164 (38.59)	**<0**.**001**
ICU admission	14 (3.1)		14 (42.42)		0 (0)	**<0**.**001**
Mortality	15 (3.3)		15 (45.45)		0 (0)	**<0**.**001**
Septic shock	19 (4.1)		19 (57.58)		0 (0)	**<0**.**001**

### The relationship between Inflammation/nutrition-based indicators and poor prognosis in PLA patients

To identify independent predictors among the inflammation/nutrition-based indices, we constructed three sequential regression models (Table 5). Model 0 provided unadjusted estimates; Model 1 adjusted for age and sex; Model 2 further incorporated variables that had shown *p* < 0.10 in the univariate screen—namely platelet count, total bilirubin, and AST—together with age and sex. The optimal cut-off values for dichotomization were determined by maximizing the Youden index (sensitivity + specificity − 1) from ROC analysis: HALP 45.5, NLR 9.42, PLR 361.8, NAR 0.62, PNI 57.88, CONUT 7.5, and IBI 1,288.67. After this stepwise selection, multivariable analysis in Model 2 identified NAR (OR 6.87, 95% CI [2.13–22.17], *p* = 0.001), CONUT (OR 1.37 per SD, *p* < 0.001), and PNI (OR 0.99 per SD, *p* = 0.038) as independent predictors of poor prognosis . In exploratory analyses using dichotomised thresholds derived from the Youden index (NAR ≥ 0.62, PNI < 57.88, CONUT ≥ 7.5, NLR ≥ 9.42), these cut points were associated with poor prognosis in univariable analysis.

**Table 5:** The univariate and multivariatelogistic regression analysis of risk factors associated with poor prognosis in patients with suppurative liver abscess. OR, odds ratio; CI, confidence interval; Model 0: unadjusted; Model 1: adjusted for age and gender; Model 2: adjusted for age, gender, Platelets, Total bilirubin, AST. For continuous variables presented as “Per SD (increased)”, the OR represents the risk associated with a one–standard deviation increment in the respective variable. Poor prognosis included either death, shock, or admission to the ICU. Bold values indicate statistical significance (*P* < 0.05).

	Model 0		Model 1		Model 2	
	OR (95% CI)	*P* value	OR (95% CI)	*P* value	OR (95% CI)	*P* value
Age	1.940 (0.942–3.995)	0.887	0.992 (0.966–1.020)	0.588	0.423 (0.186–0.959)	0.079
Sex	0.998 (0.972–1.025)	0.072	2.035 (0.966–4.286)	0.062	0.982 (0.954–1.011)	0.219
Platelets 109/L	0.993 (0.990–0.997)	**<0**.**001**	0.993 (0.989–0.996)	**<0**.**001**	0.995 (0.992–0.999)	**0**.**001**
Total bilirubin µmol/L	1.008 (1.000–1.016)	**0**.**046**	1.009 (1.001–1.017)	**0**.**036**	1.004 (0.993–1.017)	0.457
AST μ mol/L					1.001 (0.999–1.003)	0.092
HALP						
Per SD (increased)	1.000 (0.999–1.001)	0.976	1.000 (0.999–1.001)	0.967	1.000 (0.998–1.001)	0.696
<45.5	Reference		Reference		Reference	
≥45.5	0.277 (0.129–0.596)	**0**.**001**	0.236 (0.107–0.522)	**<0**.**001**	0.675 (0.263–1.729)	0.413
NLR						
Per SD (increased)	1.043 (1.014–1.073)	**0**.**004**	1.046 (1.016–1.076)	0.002	1.026 (0.993–1.060)	0.125
<9.42	Reference		Reference		Reference	
≥9.42	0.187 (0.084–0.416)	**<0**.**001**	0.178 (0.079–0.400)	**<0**.**001**	0.277 (0.115–0.657)	**0**.**004**
PLR						
Per SD (increased)	0.992 (0.988–0.997)	**0**.**001**	0.992 (0.987–0.996)	0.001	0.999 (0.994–1.004)	0.691
<361.8	Reference		Reference		Reference	
≥361.8	0.952 (0.277–3.270)	0.937	0.954 (0.275–3.303)	0.940	0.414 (0.107–1.602)	0.201
NAR						
Per SD (increased)	9.473 (3.188–28.143)	**<0**.**001**	11.010 (3.619–33.493)	**<0**.**001**	6.866 (2.127–22.167)	**0**.**001**
<0.62	Reference		Reference		Reference	
≥0.62	0.197 (0.084–0.462)	**<0**.**001**	0.188 (0.079–0.449)	**<0**.**001**	0.329 (0.126–0.859)	**0**.**023**
PNI						
Per SD (increased)	0.898 (0.853–0.946)	**<0**.**001**	0.899 (0.853–0.947)	**<0**.**001**	0.994 (0.894–0.997)	**0**.**038**
<57.88	Reference		Reference		Reference	
≥57.88	0.140 (0.033–0.588)	**0**.**007**	0.109 (0.024–0.494)	**0**.**004**	0.094 (0.016–0.537)	**0**.**008**
CONUT						
Per SD (increased)	1.516 (1.306–1.761)	**<0**.**001**	1.511 (1.300–1.757)	**<0**.**001**	1.366 (1.164–1.602)	**<0**.**001**
<7.5	Reference		Reference		Reference	
≥7.5	0.131 (0.057–0.299)	**<0**.**001**	0.128 (0.055–0.297)	**<0**.**001**	0.219 (0.089–0.540)	**<0**.**001**
IBI						
Per SD (increased)	1.000 (1.000–1.000)	0.121	1.000 (1.000–1.000)	0.139	1.000 (1.000–1.000)	0.618
<1,288.67	Reference		Reference		Reference	
≥1,288.67	0.155 (0.049–0.492)	**0**.**002**	0.150 (0.047–0.483)	**0**.**001**	0.277 (0.081–0.950)	**0**.**041**

In contrast, HALP, PLR, and IBI failed to demonstrate independent prognostic value. Following multivariable adjustment, HALP was non-significant both as a continuous variable (per SD increase, *p* = 0.696) and dichotomized at ≥ 45.5 (*p* = 0.413). Similarly, PLR showed no significant association in either continuous (*p* = 0.691 in Model 2) or dichotomized form (≥ 361.8, *p* = 0.201), and IBI was non-significant across all model specifications (continuous: *p* = 0.618; ≥ 1,288.67: *p* = 0.041 in Model 2 but not independent after full adjustment).

### Prognostic value of each Inflammation/nutrition-based index and platelets

The prognostic value of each inflammation/nutrition-based index and platelets was evaluated using ROC analysis. The results are summarized in Fig. 1. The AUC values for HALP, PLR, NLR, NAR, PNI, CONUT, IBI, and PLT were 0.566 (95% CI [0.444–0.688], *p* = 0.214), 0.704 (95% CI [0.597–0.811], *p* < 0.001), 0.669 (95% CI [0.562–0.775], *p* = 0.001), 0.675 (95% CI [0.571–0.780], *p* = 0.001), 0.757 (95% CI [0.549–0.865], *p* < 0.001), 0.803 (95% CI [0.727–0.879], *p* < 0.001), 0.711(95% CI [0.604–0.818], *p* = 0.003) and 0.740 (95% CI [0.629–0.851], *p* < 0.001), respectively.

**Figure 1:**
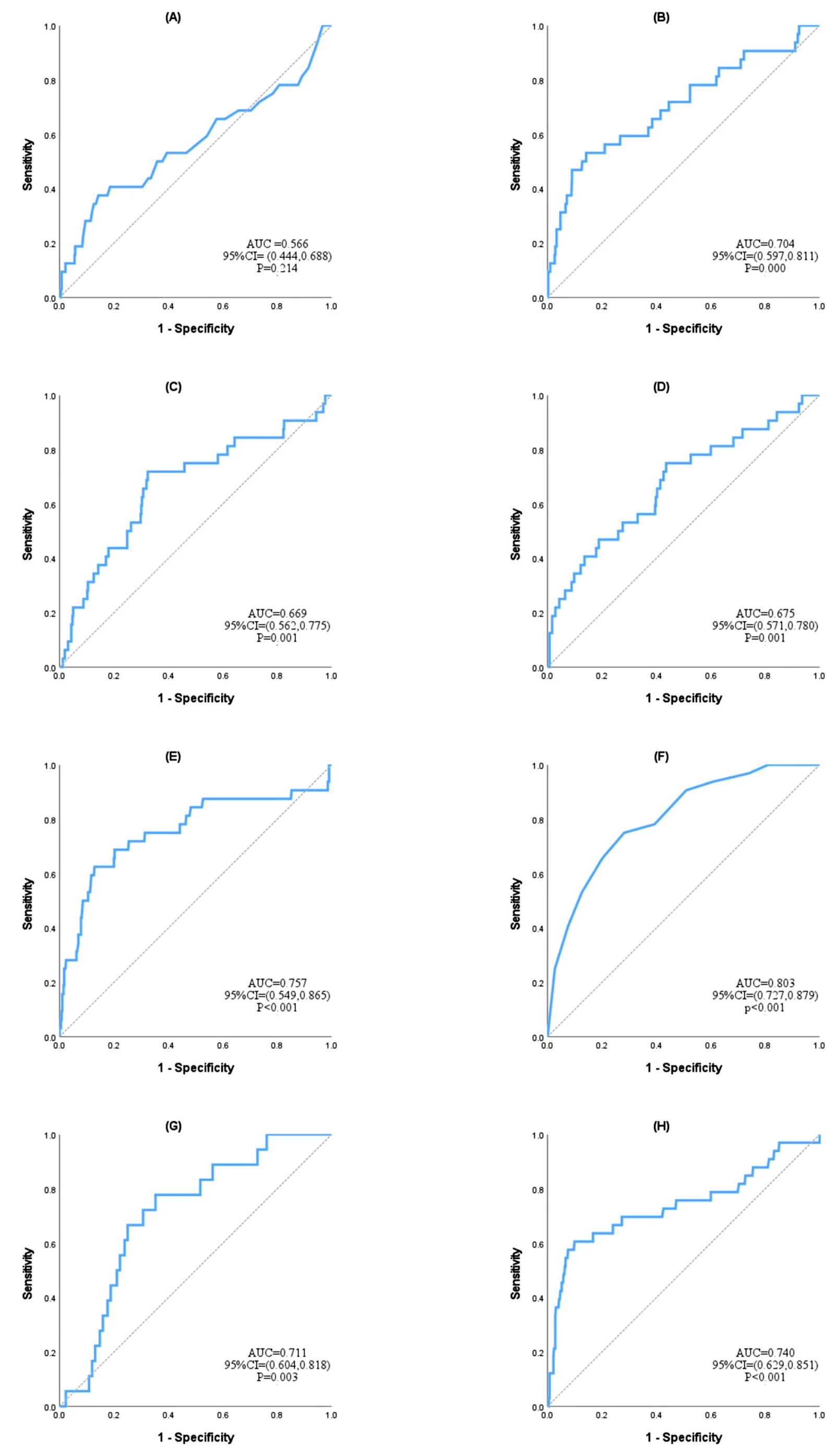
ROC analysis of each Inflammation/nutrition-based index and platelets for the poor prognosis. (A) Hemoglobin, albumin, lymphocyte, and platelet (HALP) score; (B) platelet-to-lymphocyte ratio (PLR); (C) Neutrophil-to-lymphocyte ratio (NLR); (D) Neutrophil-to-albumin ratio (NAR); (E) Prognostic nutritional index (PNI); (F) Controlling nutritional status (CONUT) score; (G) Inflammatory burden index (IBI); (H) Platelet count (PLT). AUC, The area under the curve. poor prognosis included either death, shock, or admission to the ICU.

### The construction and assessment of joint prediction model

Based on the predefined selection criteria and collinearity assessment, platelet count (PLT) and CONUT score were identified as the optimal predictors for the combined model. Bootstrap validation confirmed the stability of the regression coefficients (Table S1). ROC analysis showed that the combined model yielded an AUC of 0.822 (95% CI [0.649–0.865], *p* < 0.001) (Fig. 2). The optimal cut-off value for the model’s predicted probability was 0.188, determined by the Youden index, corresponding to a sensitivity of 68.8% and a specificity of 86.9% (Table S2). In addition, to address the potential heterogeneity of the composite outcome (which included mortality, ICU admission, and septic shock), we performed a sensitivity analysis evaluating the prognostic performance of the joint prediction model for each individual component (Fig. S1).

**Figure 2:**
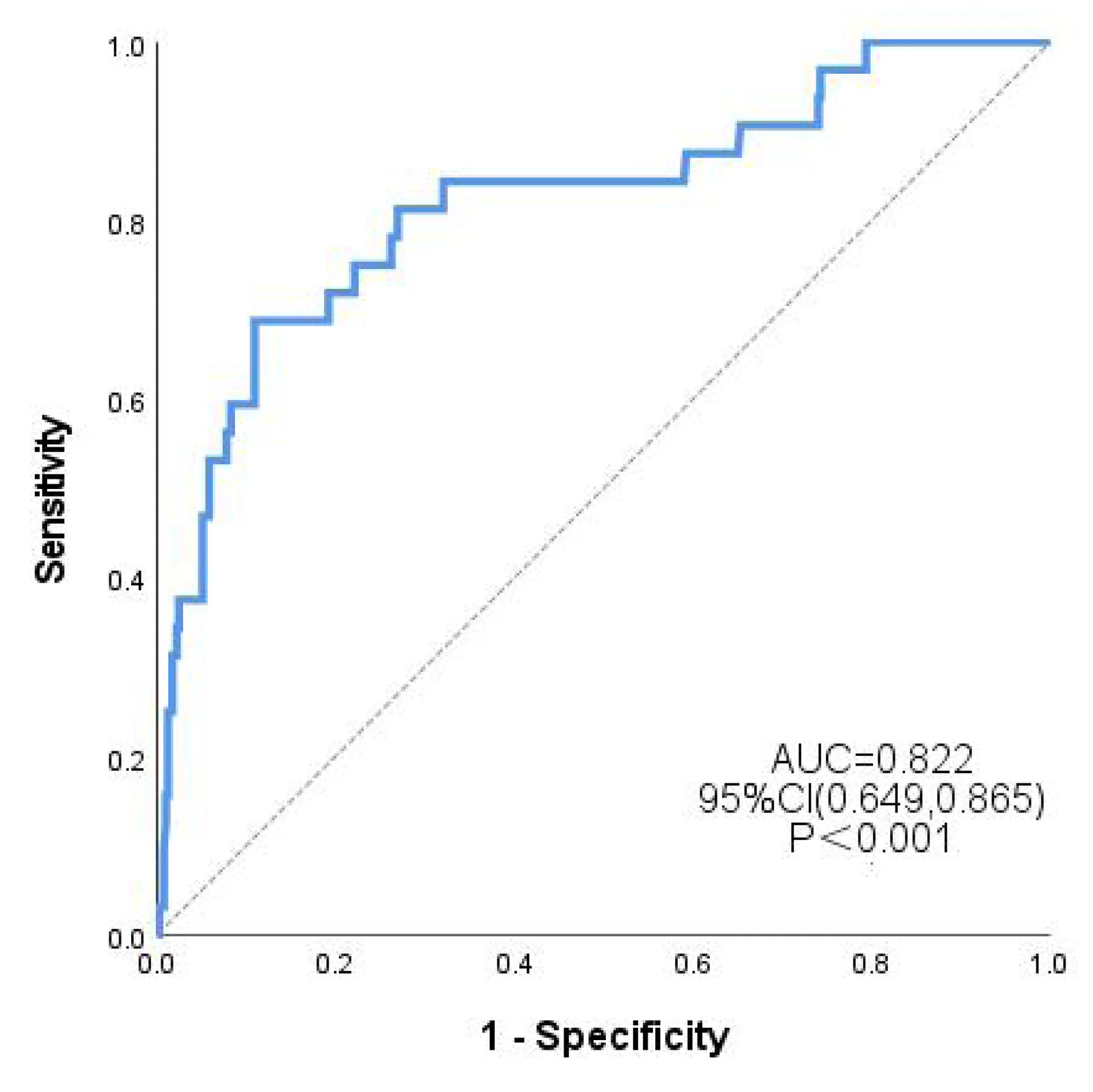
ROC curve of the joint prediction model (PLT and CONUT) for poor prognosis. AUC, The area under the curve. Poor prognosis included either death, shock, or admission to the ICU.

## Discussion

Evidence is mounting that inflammatory and nutritional indices can reliably predict outcomes across various diseases, particularly in cancer and other chronic conditions, where they have demonstrated significant prognostic value. However, the optimal marker for prognosis in PLA has yet to be established. Our study provides compelling evidence that integrated inflammation-nutrition indices, such as the NAR, PNI, and CONUT score, are superior to conventional biomarkers in identifying patients at the highest risk of poor prognosis, including mortality, septic shock, or ICU admission. Meanwhile, several key findings warrant in-depth analysis.

Patients experiencing poor prognosis exhibited marked hematologic and biochemical abnormalities, including lymphocytopenia (0.8 *vs.* 1.3  ×  10^9^/L, *p* = 0.005), anemia (103 *vs.* 116 g/L, *p* < 0.001), thrombocytopenia (74 *vs.* 238  ×  10^9^/L, *p* < 0.001), and hypoalbuminemia (25.9 *vs.* 32.1 g/L, *p* < 0.001). These abnormalities indicate a state of global physiological decompensation. The complex interplay of malnutrition and inflammation impairs tissue repair, disrupts microvascular integrity, and induces immune paralysis, thereby predisposing patients to multiorgan failure. Similar observations have been reported in sepsis research, where the combination of nutritional deficiency and inflammatory stress exacerbates organ dysfunction through dysregulated cytokine signaling and endothelial damage (Dolmatova et al., 2021).

Through univariate logistic regression analysis, it is clear that NAR is the strongest independent predictor (OR = 6.87, 95% CI [2.13–22.17]), mechanically linking neutrophil-driven inflammation with hypoalbuminemia. Drawing from established evidence in malignancies, where inflammatory-nutritional indices predict survival through tumor-promoting systemic inflammation (Sugawara et al., 2022), this dual damage promotes organ injury through oxidative stress and the collapse of tumor microenvironment pressure homeostasis—where hypoalbuminemia-driven oncotic pressure loss exacerbates interstitial edema, hypoperfusion, and subsequent reactive oxygen species (ROS) accumulation. Studies on critical diseases have shown that the neutrophil-albumin axis is an indicator of disease severity, reflecting the inflammatory burden and vascular permeability (Bao et al., 2024). In PLA, the interaction between neutrophil activation and albumin levels may exacerbate liver injury and systemic inflammation, highlighting the importance of targeting inflammation and nutritional pathways in therapeutic strategies.

Our study also uncovered several findings that merit further exploration. For instance, the lack of a significant association between CRP and poor prognosis (*p* = 0.639) is somewhat unexpected, given the widely recognized role of CRP as an acute-phase reactant in inflammatory conditions. This null finding may be attributed to several interrelated factors. First, CRP is nearly universally elevated in PLA patients due to the nature of intra-abdominal infection, reducing its discriminative power for risk stratification among patients who already have significant inflammatory burden. Second, as a retrospective study, blood samples were collected at the time of patient admission—a single time point with inherent randomness. This approach cannot account for the dynamic nature of the inflammatory response, and a single measurement may therefore not fully capture the true severity of infection. Third, a substantial proportion of patients had received prior antibiotic therapy before admission or blood sampling, which can substantially attenuate CRP levels, potentially masking the severity of the underlying infection independent of the ultimate clinical course. Furthermore, the paradoxical finding that a high dichotomized NLR (≥9.42) appeared as a protective factor (OR = 0.28) warrants careful interpretation. While NLR elevation is conventionally viewed as a marker of poor prognosis, emerging evidence suggests that the relationship between NLR and mortality in critically ill populations may be nonlinear. This is not an isolated phenomenon, a recent study (Lee et al., 2024) found that in a similarly critically ill cohort of patients with septic acute kidney injury requiring continuous renal replacement therapy, a high NLR (≥18) was unexpectedly associated with significantly lower in-hospital mortality (70.4% *vs.* 83.4%, *p* < 0.001). This counterintuitive observation may be explained by several mechanisms: first, the timing of death—patients at risk of early death exhibited significantly lower NLR at admission compared to survivors, whereas late death was associated with increasing NLR over time (Riché et al., 2015); second, therapeutic interventions such as corticosteroid administration can attenuate neutrophil counts, artificially lowering NLR independent of the underlying infectious severity (Lee et al., 2024). In our PLA cohort, the observed protective effect of high NLR may similarly reflect these complex dynamics. We therefore emphasize that this finding should be regarded as exploratory and hypothesis-generating, warranting further investigation in larger, adequately powered studies with serial NLR measurements. Additionally, the inversion of the PLR in the poor prognosis group (91.70 *vs.* 185.61, *p* < 0.001) highlights the limitations of ratio-based indices in contexts of extreme thrombocytopenia, suggesting the need for concurrent assessment of absolute platelet counts (AUC = 0.740). Furthermore, the absence of an association between abscess characteristics (size, loculation, gas presence) and outcomes indicates that factors other than abscess characteristics, such as cardiovascular comorbidities (Kearney & Knoll, 2015; Lee et al., 2021; Sharma et al., 2010; Yin et al., 2021) (9.1% *vs.* 2.4%, *p* = 0.025), may influence outcomes, potentially through mechanisms like endothelial dysfunction and microcirculatory failure. These observations emphasize the multifactorial nature of PLA and suggest that a comprehensive approach, considering both inflammatory and nutritional status, may be necessary for accurate prognostication.

Clinically, the discriminative hierarchy we established offers practical guidance for patient management. The PNI and CONUT score serve as valuable tools for identifying patients with nutritional vulnerability. The PNI, calculated using serum albumin and lymphocyte count, is a well-established index that has been shown to predict outcomes in various diseases, including cancer and chronic inflammatory conditions (Suzuki et al., 2023). Using the optimal cut-off value determined by the Youden index, our analysis further quantifies its prognostic significance in PLA, demonstrating that patients with a preserved PNI (≥57.88) exhibited a significantly reduced risk of poor prognosis (OR = 0.094, *P* = 0.008). This finding reinforces that a low PNI, indicative of malnutrition and immune suppression, can critically impair the body’s ability to fight infections and heal wounds—a particularly relevant concern in PLA, where both adequate nutrition and a robust immune response are essential for effective treatment and recovery. Similarly, the CONUT score, which combines serum albumin, total lymphocyte count, and total cholesterol levels, provides a comprehensive assessment of a patient’s nutritional status. Chronic malnutrition, as indicated by a high CONUT score, can lead to muscle wasting, reduced organ function, and impaired wound healing (Di Vincenzo et al., 2023). In our analysis, CONUT ≥ 7.5 emerged as the strongest independent risk factor for poor prognosis (OR = 4.564, *P* < 0.001), further underscoring that significant nutritional compromise critically impacts organ function and recovery. This is particularly relevant in PLA, as adequate nutrition is essential for maintaining liver function and supporting the immune system. The NAR, which combines neutrophil count and serum albumin, reflects the interplay between inflammation and nutrition. Elevated neutrophil levels indicate an active inflammatory response, while low albumin levels suggest inadequate nutritional support. This imbalance can exacerbate the inflammatory process, leading to increased tissue damage and a higher risk of complications (Liu & Chien, 2023; Zhang et al., 2024). In PLA, this imbalance can contribute to the progression of liver injury and systemic inflammation, ultimately affecting patient outcomes. Using Youden-derived cut-off values (NAR ≥ 0.62, PNI < 57.88, CONUT ≥ 7.5), we observed in exploratory analyses that these thresholds were associated with poor prognosis, which identifies patients in whom the inflammatory-nutritional imbalance has reached a critical level. However, it is important to emphasise that these cut points were optimised to our specific dataset and were not prespecified. Therefore, these dichotomised findings are exploratory and hypothesis-generating in nature, and they should not be interpreted as definitive clinical thresholds without external validation. Several other inflammatory-nutritional indices demonstrated varying degrees of clinical relevance. IBI ≥ 1,288.67 was independently associated with poor prognosis across all models (Model 2 OR = 0.277, *P* = 0.041), suggesting that the cumulative immune-inflammatory burden captured by this composite index may help identify patients requiring more aggressive intervention. In contrast, HALP and PLR showed limited clinical utility after full adjustment. HALP ≥ 45.5 lost statistical significance in the fully adjusted model (OR = 0.675, *P* = 0.413), while PLR demonstrated no significant association with poor prognosis in any model, indicating that these indices may not provide additional predictive value beyond the more robust markers identified in our study. The paradoxical finding regarding NLR ≥9.42 has been discussed above. These indices provide a multifaceted view of a patient’s condition, integrating both nutritional and inflammatory status. By using these indices, clinicians can identify patients at high risk of poor prognosis early in their disease course, allowing for timely and targeted interventions.

Building on the insights derived from individual indices, our combined prediction model demonstrated promising prognostic performance. The model integrates PLT and CONUT with a stable AUC value of 0.822 (Fig. 2). A key strength of this model is its clinical applicability, facilitated by an easily interpretable cut-off value. Using the Youden index, we established an optimal predicted probability threshold of ≥0.188 for identifying high-risk patients, which provides a sensitivity of 68.8% and a specificity of 86.9%. At the point of admission, a patient whose calculated probability exceeds this cut-off can be objectively flagged as “high-risk.” This simple, bedside tool carries immediate clinical implications, prompting clinicians to initiate a bundle of proactive measures such as more frequent monitoring for early signs of deterioration, consideration of intensified nutritional support, and closer observation of coagulation status. By enabling objective and early identification of high-risk individuals, this cut-off can guide clinical decision-making and optimize resource allocation. The rationale for this combined model is further supported by previous research, which has highlighted the value of integrating multiple biomarkers to capture the complex interplay of inflammation and nutrition in various disease contexts. For instance, studies in cancer and chronic inflammatory conditions have shown that combining nutritional and inflammatory parameters can enhance risk stratification compared to single markers (Jiao et al., 2023; Sugawara et al., 2022; Xu et al., 2024). Our findings align with this body of work, emphasizing that no single index can fully encapsulate the multifaceted pathophysiology of PLA.Consistent with this paradigm, the superior performance of our joint model over any single index can be attributed to its ability to capture two complementary dimensions of patient pathophysiology. On one hand, PLT reflects not only the systemic inflammatory response and its interaction with the coagulation system but also the body’s immune defense capacity (Li et al., 2022). On the other hand, the CONUT score, calculated from serum albumin, total cholesterol, and total lymphocyte count, provides a comprehensive assessment of the patient’s nutritional and immunological reserve (Sharma et al., 2010). The synergy of this model lies in its ability to simultaneously capture the acute inflammatory-coagulative response (PLT) and the pre-existing or developing state of nutritional-immune depletion (CONUT), which are both critical determinants of clinical trajectory in severe infections. This aligns with findings in other disease contexts, where integrated models that combine inflammatory and nutritional markers have consistently outperformed single markers by accounting for the complex interplay between these systems (Chen et al., 2026; Xu et al., 2022).

The clinical significance of our findings lies in the model’s practicality and potential for superior performance. The constituent variables of our joint model (PLT and CONUT) are all derived from routine, low-cost blood tests, making it readily implementable even in resource-limited settings. While established scores like APACHE II or SOFA are also prognostic, they are more complex to calculate and are often used in critically ill, ICU-level patients. Our model, in contrast, provides a powerful tool for early risk assessment at the time of admission, potentially identifying high-risk patients before they progress to organ failure. Future studies directly comparing our model’s predictive accuracy against these traditional scores in the PLA population are warranted to confirm its incremental value.

While our results are encouraging, this study has several limitations. First, its retrospective, single-center design inherits the risk of selection bias and limits the generalizability of our findings to other populations. Second, the relatively small sample size of the poor prognosis group (*n* = 33) may have affected the stability of our multivariate estimates.Specifically, the wide confidence interval for the NAR per-SD odds ratio (OR 6.87, 95% CI [2.13–22.17]) may suggest potential instability in this estimate, which could be attributable to the limited number of events and the associated low events-per-variable ratio (EPV). This caution should be considered when interpreting this finding. We suggest that future multicenter studies with larger sample sizes and a greater number of adverse outcome events be conducted to further validate and strengthen the stability of this estimate While Bootstrap validation confirmed the internal stability of our simplified model, external validation in independent, multicenter cohorts is necessary to confirm the generalizability of our findings before clinical implementation. The limited number of events also precludes meaningful subgroup analyses (*e.g.*, by pathogen type or specific comorbidities like diabetes). Third, we excluded patients with conditions like cirrhosis and CHF, which means our conclusions may not apply to these important subpopulations. Fourth, the cut-off values for inflammation–nutrition indices (*e.g.*, NAR ≥ 0.62, PNI < 57.88, CONUT ≥ 7.5) were derived using the Youden index to maximise sensitivity and specificity in our cohort. Because these thresholds were data-driven and not prespecified, they are subject to optimistic bias. We have therefore designated all dichotomised analyses based on these cut points as exploratory only. External validation in independent cohorts is required before these thresholds can be considered for clinical application. Furthermore, the composite outcome used in this study combined mortality (a hard endpoint) with ICU admission and septic shock, which are inherently threshold-driven decisions. Although our sensitivity analysis supported the overall feasibility of this composite measure, we acknowledge that some degree of heterogeneity may exist among its individual components, and this should be considered when interpreting the primary findings. Finally, the absence of serial measurements prevents us from assessing the dynamic changes of these indices, which might offer even greater prognostic insight than single-time-point values.

Despite these limitations, our findings robustly demonstrate the prognostic value of inflammation-nutrition indices in PLA and establish a novel, high-accuracy prediction model. This sets a foundation for improved clinical risk assessment.

## Conclusion

In conclusion, this study provides compelling evidence that inflammation- and nutrition-based indices, including the NAR, PNI, and CONUT score, are powerful predictors of poor prognosis in PLA. These readily available indices provide a cost-efficient, non-invasive modality for severity assessment and outcome prediction, helping clinicians identify high-risk patients early and implement targeted interventions. A key highlight is the development and internal validation of a joint prediction model incorporating PLT and CONUT, which demonstrated reasonable prognostic accuracy with AUC of 0.822. This integrated model provides a comprehensive assessment of a patient’s condition by combining nutritional and inflammatory status, offering a practical tool for early risk stratification and guiding clinical decision-making. Nevertheless, well-designed prospective multicenter studies are warranted to externally validate these findings and to further refine the risk stratification algorithm for broader clinical application.

##  Supplemental Information

10.7717/peerj.21852/supp-1 Supplemental Information 1 Raw data

10.7717/peerj.21852/supp-2 Supplemental Information 2 STROBE Documentation

10.7717/peerj.21852/supp-3 Supplemental Information 3 Codebook

10.7717/peerj.21852/supp-4 Supplemental Information 4 The result of the Bootstrap validation A. Variables in the Equation; B. Bootstrap for Variables in the Equation

10.7717/peerj.21852/supp-5 Supplemental Information 5 ROC coordinates of the joint prediction model

10.7717/peerj.21852/supp-6 Supplemental Information 6 Model performance metrics

10.7717/peerj.21852/supp-7 Supplemental Information 7 Sensitivity analysis of individual components of the composite outcome A.Sensitivity analysis of mortality (B) Sensitivity analysis of ICU admission. (C) Sensitivity analysis of septic shock

10.7717/peerj.21852/supp-8 Supplemental Information 8 Model performance metrics. A. optimism-corrected AUC; B. Decision Curve; C. Calibration Plot

## Additional Information and Declarations

### Data Availability

The following information was supplied regarding data availability: The raw measurements are available in the Supplemental Files.
