# Supplementary material for: Inflammatory and nutritional indices as predictors of poor prognosis in pyogenic liver abscesses: a retrospective cohort study
Source: PeerJ. 2026 Oct 27;14:e21852. doi: 10.7717/peerj.21852 (PMC13721881; doi:10.7717/peerj.21852)

**Supplementary Figure 1 Sensitivity analysis of individual components of the composite outcome**
**A.Sensitivity analysis of mortality**

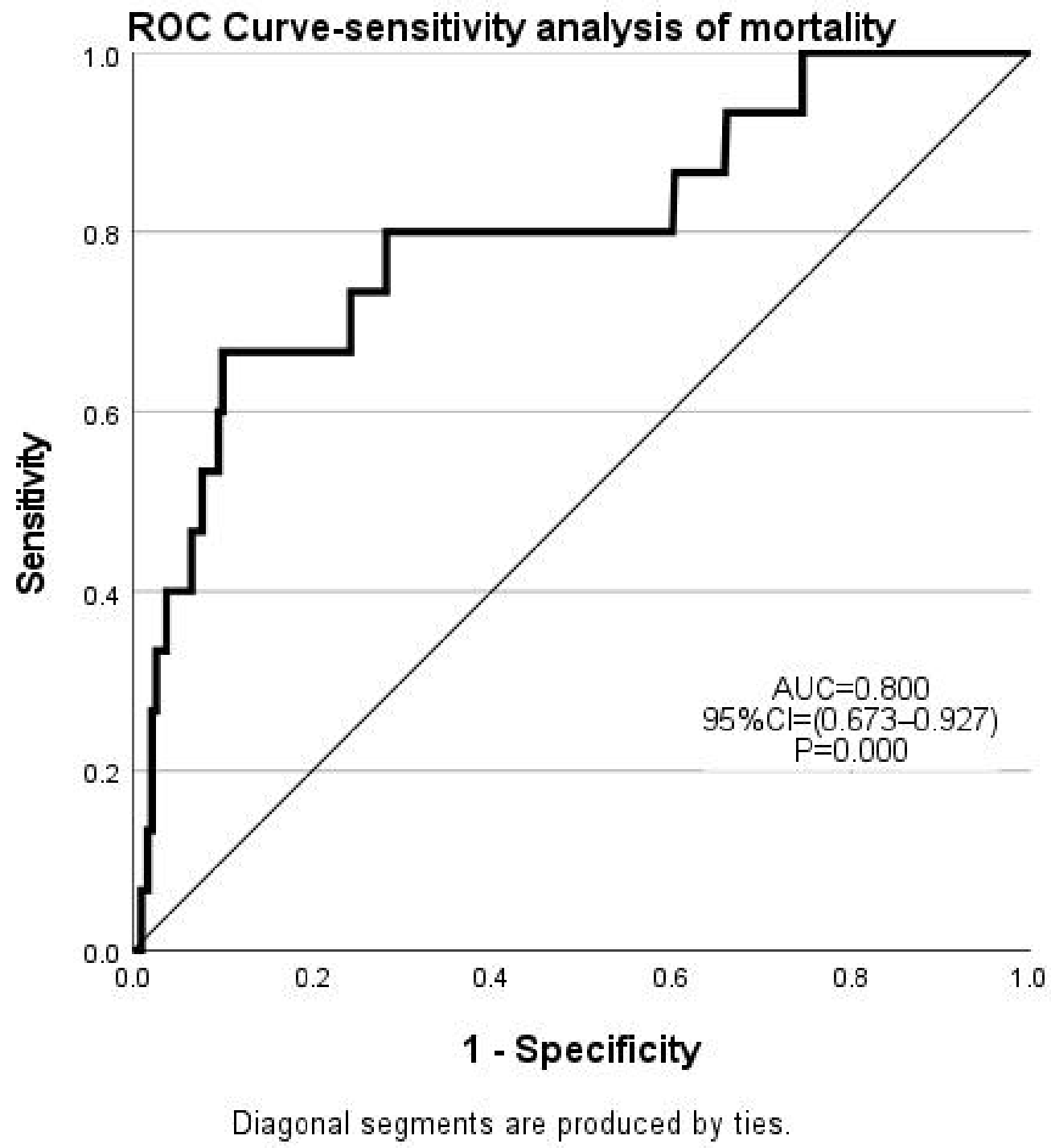


**B. Sensitivity analysis of ICU admission**

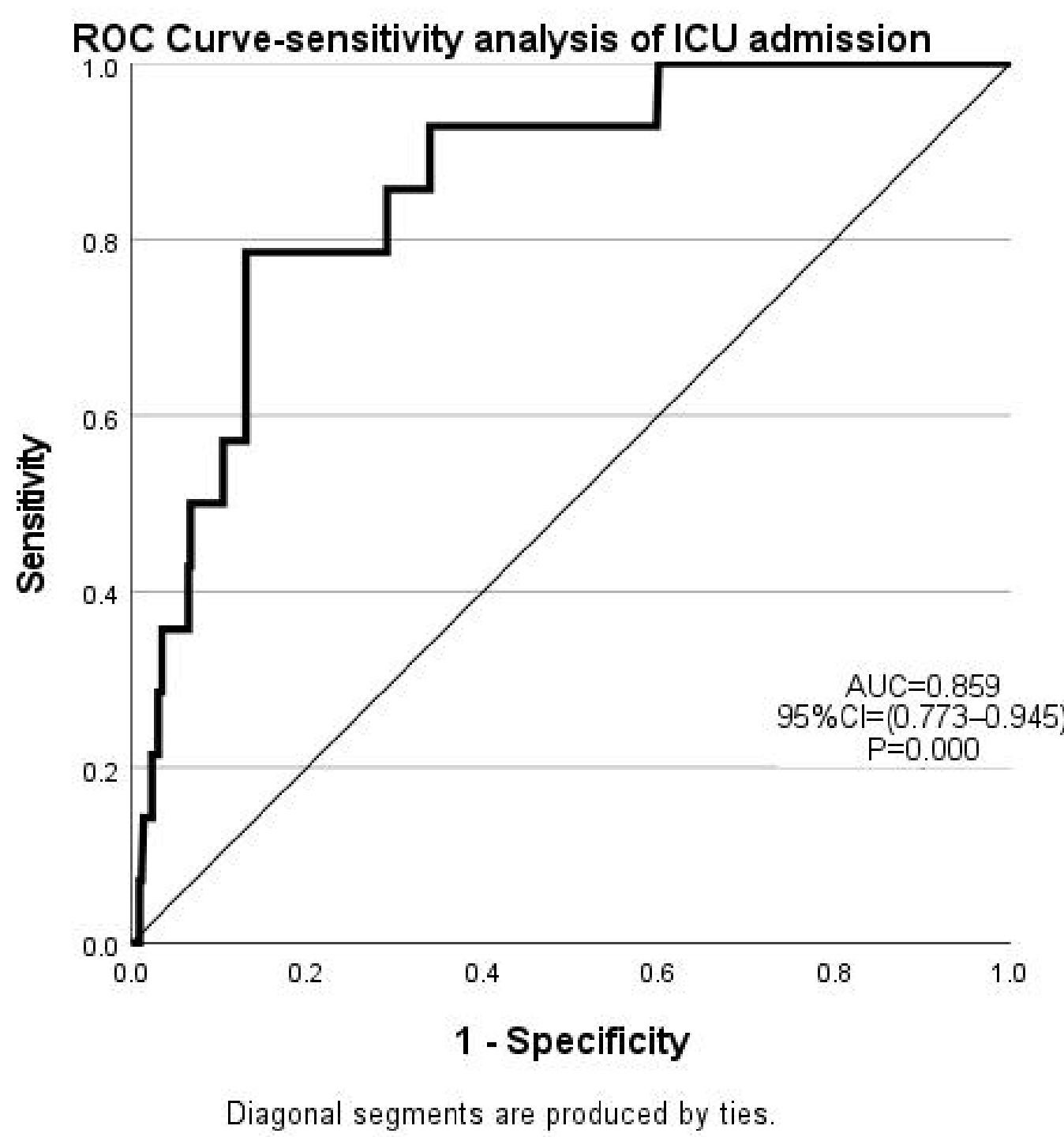

**C. Sensitivity analysis of septic shock**

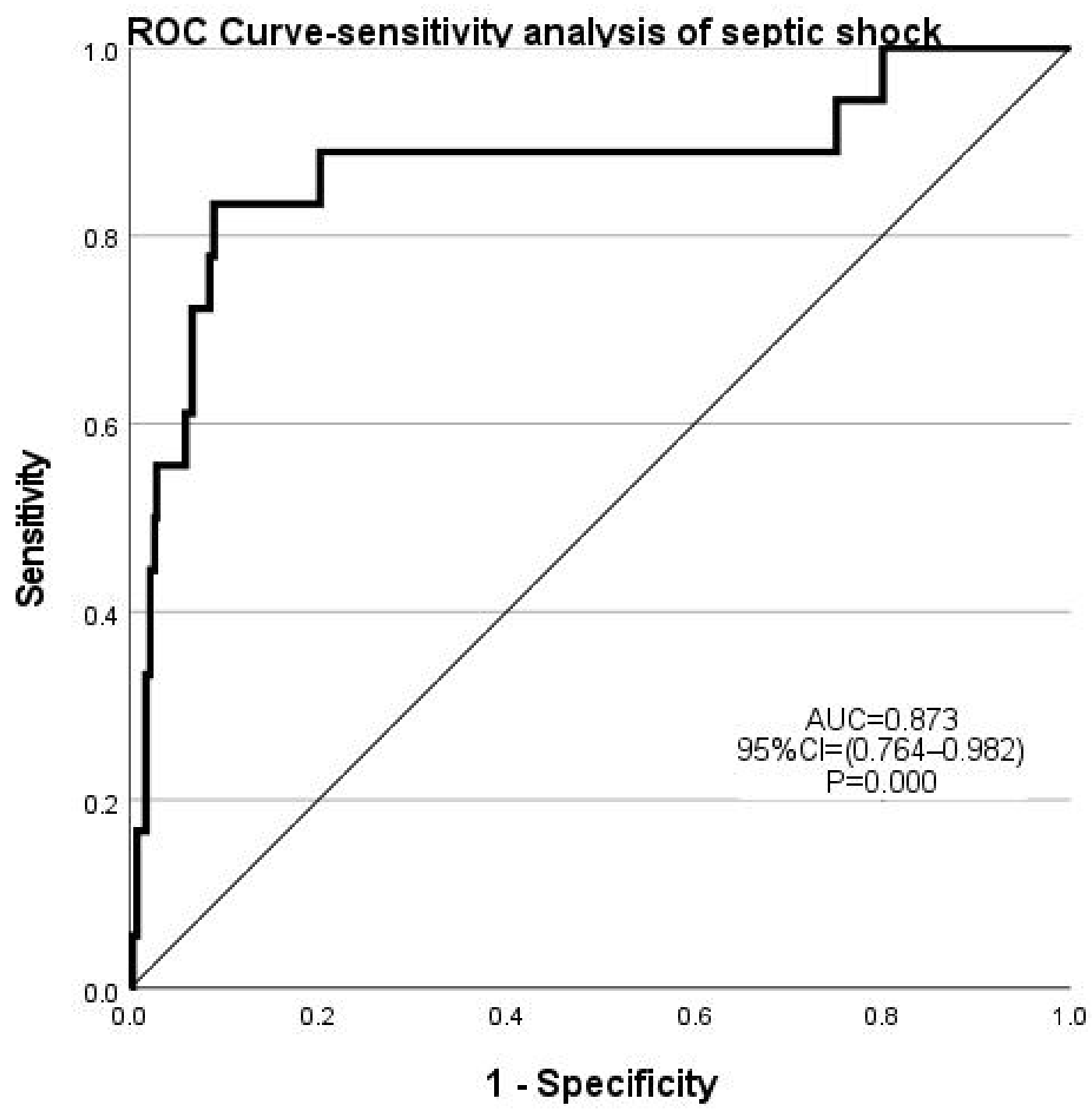

Supplement: Supplementary file 1 [file peerj-14-21852-s007.pdf]
