# Supplementary material for: Inflammatory and nutritional indices as predictors of poor prognosis in pyogenic liver abscesses: a retrospective cohort study
Source: PeerJ. 2026 Oct 27;14:e21852. doi: 10.7717/peerj.21852 (PMC13721881; doi:10.7717/peerj.21852)

Supplementary Figure 2. Model performance metrics

A. optimism-corrected AUC

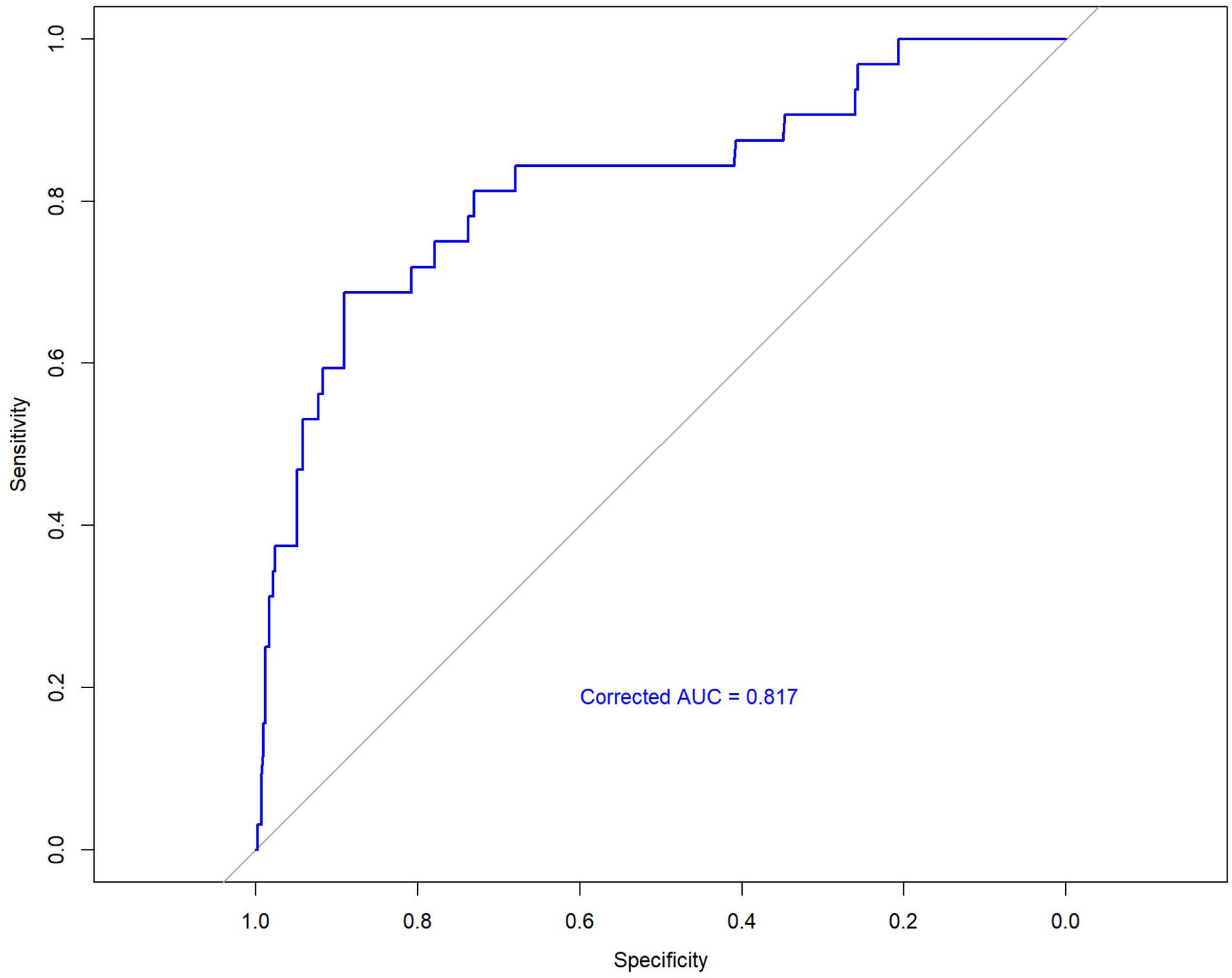

B. Decision Curve

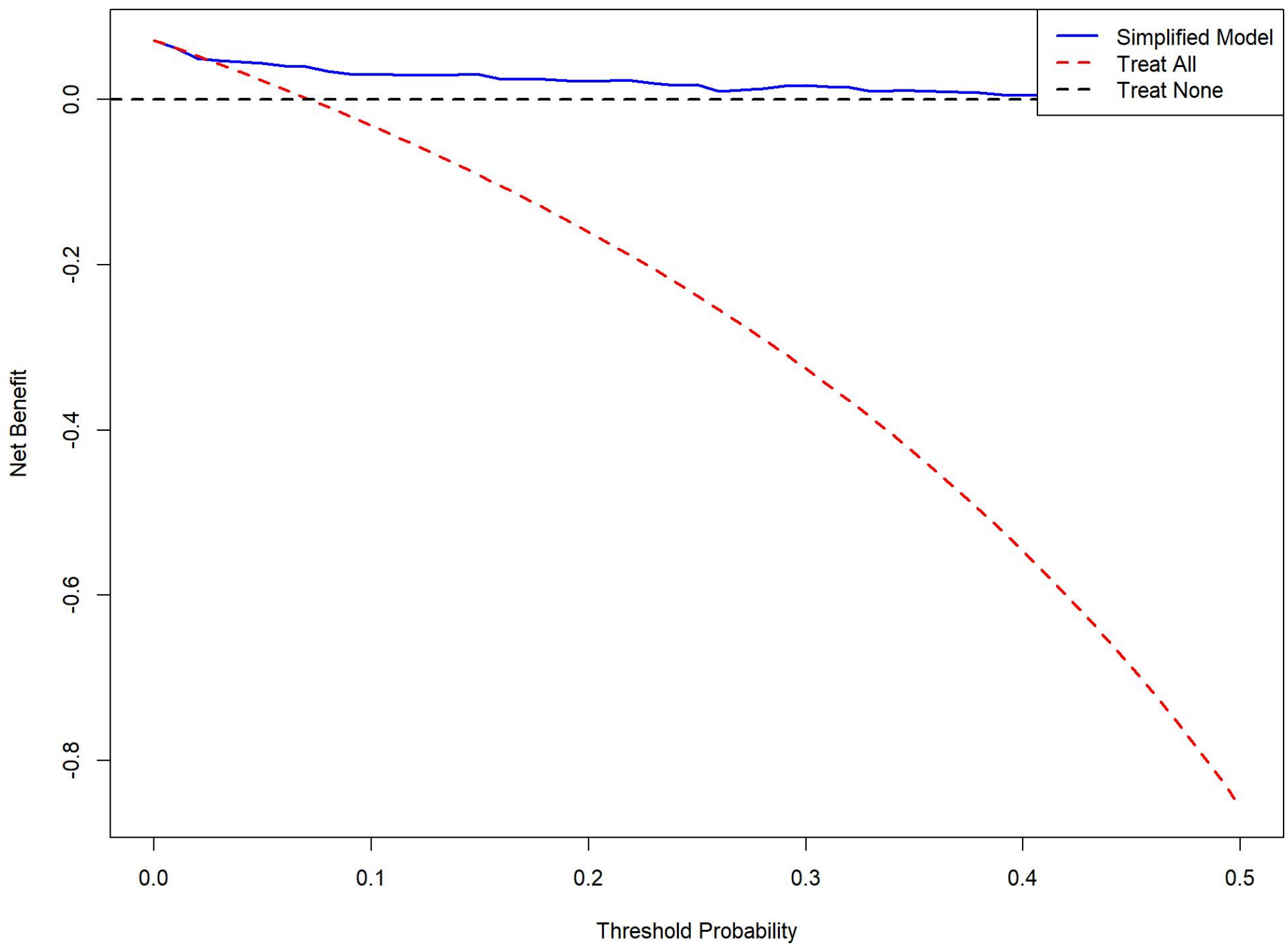

C. Calibration Plot

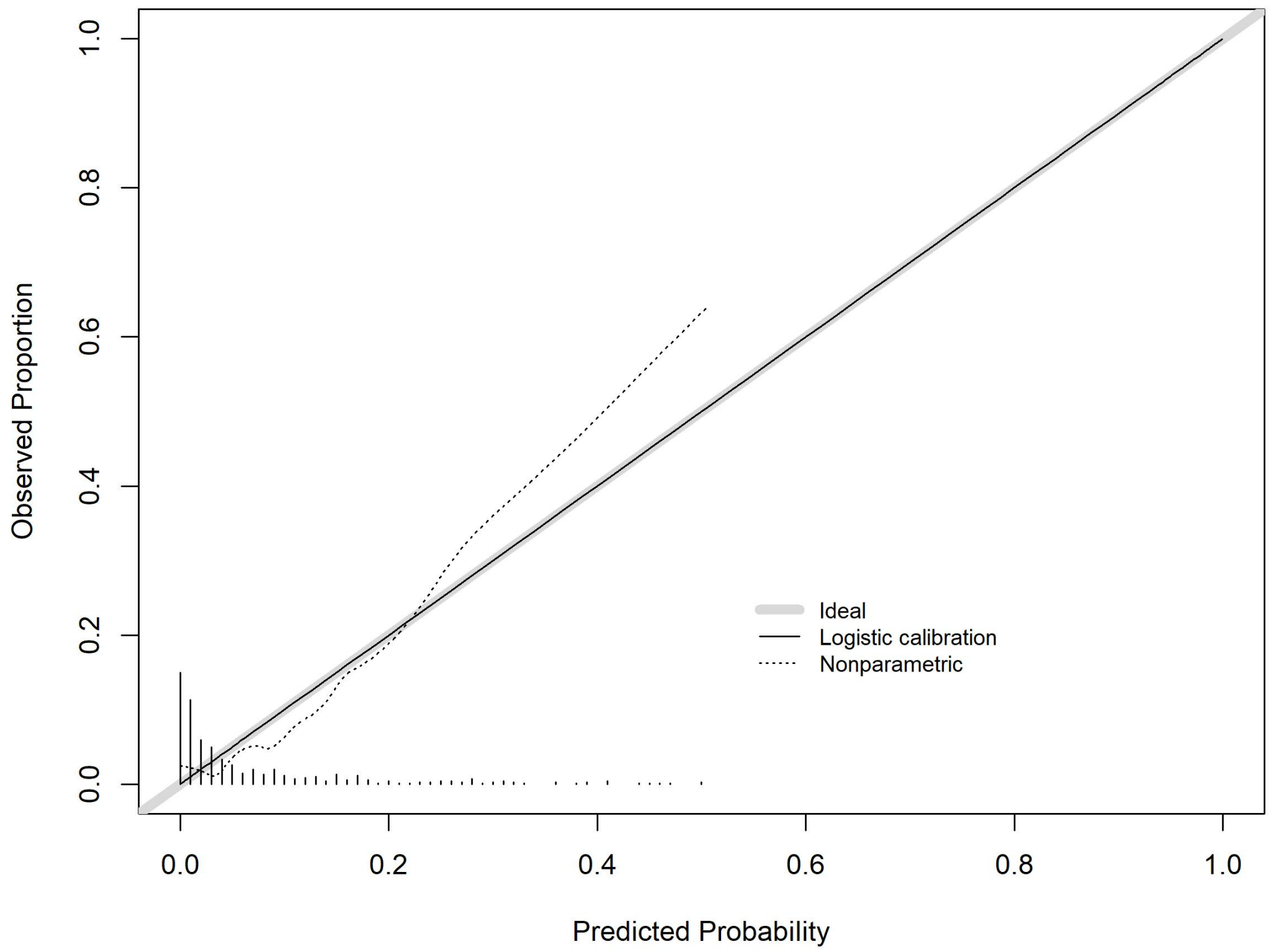

Supplement: Supplementary file 2 [file peerj-14-21852-s008.pdf]
