# Supplementary material for: Inflammatory and nutritional indices as predictors of poor prognosis in pyogenic liver abscesses: a retrospective cohort study
Source: PeerJ. 2026 Oct 27;14:e21852. doi: 10.7717/peerj.21852 (PMC13721881; doi:10.7717/peerj.21852)
Supplement: Supplementary file 3 [file peerj-14-21852-s002.docx]

STROBE Statement—checklist of items that should be included in reports of observational studies

|  | Item No. | Recommendation | Page  No. | Relevant text from manuscript |
| --- | --- | --- | --- | --- |
| **Title and abstract** | 1 | (*a*) Indicate the study’s design with a commonly used term in the title or the abstract | 1 | A retrospective cohort study |
|  |  | (*b*) Provide in the abstract an informative and balanced summary of what was done and what was found | 1 | Abstract summarizes objective, methods, key results and conclusion |
| Introduction | | | |  |
| Background/rationale | 2 | Explain the scientific background and rationale for the investigation being reported | 2 | Introduction explains PLA mortality, heterogeneity in prognosis, and rationale for using inflammation/nutrition indices |
| Objectives | 3 | State specific objectives, including any prespecified hypotheses | 3 | “This study aims to... validate and compare a comprehensive panel of inflammation-nutrition indices...” |
| Methods | | | |  |
| Study design | 4 | Present key elements of study design early in the paper | 3 | Methods: “This retrospective study analyzed a consecutive series of 458 patients with PLA...” |
| Setting | 5 | Describe the setting, locations, and relevant dates, including periods of recruitment, exposure, follow-up, and data collection | 3 | “Tongji Hospital, Wuhan, China; October 2011 to June 2021” |
| Participants | 6 | (*a*) *Cohort study*—Give the eligibility criteria, and the sources and methods of selection of participants. Describe methods of follow-up  *Case-control study*—Give the eligibility criteria, and the sources and methods of case ascertainment and control selection. Give the rationale for the choice of cases and controls  *Cross-sectional study*—Give the eligibility criteria, and the sources and methods of selection of participants | 3 | Inclusion: imaging-confirmed PLA, age ≥18, complete data. Exclusion: amebic abscess, malignancy, cirrhosis, etc. |
|  |  | (*b*) *Cohort study*—For matched studies, give matching criteria and number of exposed and unexposed  *Case-control study*—For matched studies, give matching criteria and the number of controls per case | - | - |
| Variables | 7 | Clearly define all outcomes, exposures, predictors, potential confounders, and effect modifiers. Give diagnostic criteria, if applicable | 3-4 | Poor prognosis = death/ICU/shock; predictors = NAR, PNI, CONUT; confounders = age, sex, comorbidities |
| Data sources/ measurement | 8* | For each variable of interest, give sources of data and details of methods of assessment (measurement). Describe comparability of assessment methods if there is more than one group | *4* | *“Data extracted from electronic medical records including labs, imaging, microbiology, treatments…”* |
| Bias | 9 | Describe any efforts to address potential sources of bias | 14 | Discussed: retrospective design, selection bias, excluded cirrhosis/CHF patients |
| Study size | 10 | Explain how the study size was arrived at | - | Not explicitly stated; 458 consecutive cases included |

Continued on next page

| Quantitative variables | 11 | Explain how quantitative variables were handled in the analyses. If applicable, describe which groupings were chosen and why | 5 | Continuous variables: mean±SD or median(IQR); categorized thresholds used (e.g., PNI<57.88) |
| --- | --- | --- | --- | --- |
| Statistical methods | 12 | (*a*) Describe all statistical methods, including those used to control for confounding | 5 | Multivariable logistic regression adjusted for age, sex, AST, TBIL, platelets |
|  |  | (*b*) Describe any methods used to examine subgroups and interactions | 12 | Subgroups: septic shock, ICU admission, thrombocytopenia |
|  |  | (*c*) Explain how missing data were addressed | - | Not explicitly stated; inclusion required complete data |
|  |  | (*d*) *Cohort study*—If applicable, explain how loss to follow-up was addressed  *Case-control study*—If applicable, explain how matching of cases and controls was addressed  *Cross-sectional study*—If applicable, describe analytical methods taking account of sampling strategy | - | - |
|  |  | (*e*) Describe any sensitivity analyses | - | - |
| Results | | | | |
| Participants | 13* | (a) Report numbers of individuals at each stage of study—eg numbers potentially eligible, examined for eligibility, confirmed eligible, included in the study, completing follow-up, and analysed | 6 | 458 included, 33 poor outcomes |
|  |  | (b) Give reasons for non-participation at each stage | 3 | Excluded due to incomplete data, non-pyogenic abscess, malignancy, etc. |
|  |  | (c) Consider use of a flow diagram | - | - |
| Descriptive data | 14* | (a) Give characteristics of study participants (eg demographic, clinical, social) and information on exposures and potential confounders | 6-7 | Table 1: demographics, symptoms, vitals, comorbidities |
|  |  | (b) Indicate number of participants with missing data for each variable of interest | - | - |
|  |  | (c) *Cohort study*—Summarise follow-up time (eg, average and total amount) | - | - |
| Outcome data | 15* | *Cohort study*—Report numbers of outcome events or summary measures over time | *-* | *-* |
|  |  | *Case-control study—*Report numbers in each exposure category, or summary measures of exposure | *-* | *-* |
|  |  | *Cross-sectional study—*Report numbers of outcome events or summary measures | *-* | *-* |
| Main results | 16 | (*a*) Give unadjusted estimates and, if applicable, confounder-adjusted estimates and their precision (eg, 95% confidence interval). Make clear which confounders were adjusted for and why they were included | 10 | Table 5: ORs for NAR (6.87), PNI (0.99), CONUT (1.37); adjusted for age, sex, AST, TBIL, platelets |
|  |  | (*b*) Report category boundaries when continuous variables were categorized | 10 | “PNI<57.88, NAR≥0.62, NLR≥9.42” |
|  |  | (*c*) If relevant, consider translating estimates of relative risk into absolute risk for a meaningful time period | - | Not provided |

Continued on next page

| Other analyses | 17 | Report other analyses done—eg analyses of subgroups and interactions, and sensitivity analyses | 11 | “Joint model combining PNI, IBI, CONUT, and platelet count achieved AUC=0.912” |
| --- | --- | --- | --- | --- |
| Discussion | | | | |
| Key results | 18 | Summarise key results with reference to study objectives | 15 | “NAR, PNI, and CONUT are independent predictors... joint model AUC=0.912” |
| Limitations | 19 | Discuss limitations of the study, taking into account sources of potential bias or imprecision. Discuss both direction and magnitude of any potential bias | 14-15 | “retrospective design, single-center, small adverse events (n=33), excluded cirrhosis/CHF, no serial measurements” |
| Interpretation | 20 | Give a cautious overall interpretation of results considering objectives, limitations, multiplicity of analyses, results from similar studies, and other relevant evidence | 13-15 | Discussed CRP non-significance, NLR paradox, need for prospective validation |
| Generalisability | 21 | Discuss the generalisability (external validity) of the study results | 15 | “single-center, excluded specific populations; multicenter validation warranted” |
| Other information | |  | | |
| Funding | 22 | Give the source of funding and the role of the funders for the present study and, if applicable, for the original study on which the present article is based | Title page | Funding information provided on the title page. |

*Give information separately for cases and controls in case-control studies and, if applicable, for exposed and unexposed groups in cohort and cross-sectional studies.

**Note:** An Explanation and Elaboration article discusses each checklist item and gives methodological background and published examples of transparent reporting. The STROBE checklist is best used in conjunction with this article (freely available on the Web sites of PLoS Medicine at http://www.plosmedicine.org/, Annals of Internal Medicine at http://www.annals.org/, and Epidemiology at http://www.epidem.com/). Information on the STROBE Initiative is available at www.strobe-statement.org.
