# Supplementary material for: Inflammatory and nutritional indices as predictors of poor prognosis in pyogenic liver abscesses: a retrospective cohort study
Source: PeerJ. 2026 Oct 27;14:e21852. doi: 10.7717/peerj.21852 (PMC13721881; doi:10.7717/peerj.21852)
Supplement: Supplementary file 4 [file peerj-14-21852-s004.docx]

**Supplementary Table 1 The result of the Bootstrap validation**

| **A.Variables in the Equation** | | | | | | | |
| --- | --- | --- | --- | --- | --- | --- | --- |
|  | | B | S.E. | Wald | df | Sig. | Exp(B) |
| Step 1 | Plateletcount109L | -.006 | .002 | 8.578 | 1 | .003 | .994 |
|  | CONUT | .327 | .081 | 16.519 | 1 | .000 | 1.387 |
|  | Constant | -3.883 | .873 | 19.799 | 1 | .000 | .021 |

| **B.Bootstrap for Variables in the Equation** | | | | | | | | | |
| --- | --- | --- | --- | --- | --- | --- | --- | --- | --- |
| Bootstrap^a^ | | | | | | | | | |
|  | |  |  | | Bias | Std. Error | Sig. (2-tailed) | 95% Confidence Interval | |
|  | |  | B | |  |  |  | Lower | Upper |
| Step 1 | Plateletcount109L | | | -.006 | .000 | .003 | .016 | -.013 | -.002 |
|  | CONUT | | | .327 | .013 | .082 | .001 | .197 | .525 |
|  | Constant | | | -3.883 | -.091 | .862 | .001 | -5.782 | -2.401 |
| a. Unless otherwise noted, bootstrap results are based on 1000 stratified bootstrap samples | | | | | | | | | |
