# Supplementary material for: Inflammatory and nutritional indices as predictors of poor prognosis in pyogenic liver abscesses: a retrospective cohort study
Source: PeerJ. 2026 Oct 27;14:e21852. doi: 10.7717/peerj.21852 (PMC13721881; doi:10.7717/peerj.21852)
Supplement: Supplementary file 5 [file peerj-14-21852-s005.docx]

**Supplementary Table 2 ROC coordinates of the joint prediction model**

| Coordinates of the Curve | | |
| --- | --- | --- |
| Test Result Variable(s): Predicted probability | | |
| Positive if Greater Than or Equal Toa | Sensitivity | 1 - Specificity |
| .0000000 | 1.000 | 1.000 |
| .0003531 | 1.000 | .998 |
| .0006871 | 1.000 | .995 |
| .0009338 | 1.000 | .993 |
| .0009822 | 1.000 | .990 |
| .0011219 | 1.000 | .988 |
| .0012541 | 1.000 | .985 |
| .0013461 | 1.000 | .983 |
| .0014399 | 1.000 | .981 |
| .0015988 | 1.000 | .978 |
| .0018307 | 1.000 | .976 |
| .0019474 | 1.000 | .973 |
| .0020286 | 1.000 | .971 |
| .0021114 | 1.000 | .968 |
| .0021446 | 1.000 | .966 |
| .0021514 | 1.000 | .964 |
| .0022729 | 1.000 | .961 |
| .0024321 | 1.000 | .959 |
| .0025999 | 1.000 | .956 |
| .0028927 | 1.000 | .954 |
| .0030585 | 1.000 | .951 |
| .0030785 | 1.000 | .949 |
| .0031839 | 1.000 | .947 |
| .0032840 | 1.000 | .944 |
| .0034349 | 1.000 | .942 |
| .0035989 | 1.000 | .939 |
| .0036345 | 1.000 | .937 |
| .0037241 | 1.000 | .934 |
| .0038428 | 1.000 | .932 |
| .0038833 | 1.000 | .930 |
| .0038946 | 1.000 | .927 |
| .0039402 | 1.000 | .925 |
| .0039763 | 1.000 | .922 |
| .0040533 | 1.000 | .920 |
| .0041314 | 1.000 | .917 |
| .0041574 | 1.000 | .915 |
| .0042335 | 1.000 | .913 |
| .0043125 | 1.000 | .910 |
| .0043827 | 1.000 | .905 |
| .0044517 | 1.000 | .903 |
| .0045078 | 1.000 | .900 |
| .0045565 | 1.000 | .898 |
| .0045985 | 1.000 | .896 |
| .0046521 | 1.000 | .893 |
| .0046937 | 1.000 | .891 |
| .0047073 | 1.000 | .888 |
| .0047530 | 1.000 | .886 |
| .0047976 | 1.000 | .881 |
| .0048618 | 1.000 | .876 |
| .0049849 | 1.000 | .874 |
| .0051275 | 1.000 | .871 |
| .0052252 | 1.000 | .869 |
| .0053563 | 1.000 | .867 |
| .0054894 | 1.000 | .864 |
| .0055587 | 1.000 | .862 |
| .0056134 | 1.000 | .859 |
| .0056500 | 1.000 | .857 |
| .0057184 | 1.000 | .854 |
| .0057709 | 1.000 | .852 |
| .0058084 | 1.000 | .850 |
| .0058306 | 1.000 | .847 |
| .0058503 | 1.000 | .845 |
| .0059575 | 1.000 | .842 |
| .0061662 | 1.000 | .840 |
| .0063256 | 1.000 | .837 |
| .0065088 | 1.000 | .835 |
| .0066673 | 1.000 | .833 |
| .0067451 | 1.000 | .830 |
| .0069162 | 1.000 | .828 |
| .0070922 | 1.000 | .825 |
| .0071777 | 1.000 | .823 |
| .0072243 | 1.000 | .820 |
| .0072991 | 1.000 | .818 |
| .0073959 | 1.000 | .816 |
| .0074835 | 1.000 | .813 |
| .0075468 | 1.000 | .811 |
| .0076011 | 1.000 | .808 |
| .0076522 | 1.000 | .806 |
| .0076947 | 1.000 | .803 |
| .0077654 | 1.000 | .801 |
| .0078229 | 1.000 | .799 |
| .0078491 | 1.000 | .796 |
| .0079406 | 1.000 | .794 |
| .0080400 | .969 | .794 |
| .0080884 | .969 | .791 |
| .0081864 | .969 | .789 |
| .0083391 | .969 | .786 |
| .0084473 | .969 | .784 |
| .0085635 | .969 | .782 |
| .0086936 | .969 | .779 |
| .0088264 | .969 | .777 |
| .0089408 | .969 | .774 |
| .0090511 | .969 | .772 |
| .0092155 | .969 | .769 |
| .0093664 | .969 | .767 |
| .0095179 | .969 | .765 |
| .0096558 | .969 | .762 |
| .0097160 | .969 | .760 |
| .0097227 | .969 | .757 |
| .0097791 | .969 | .755 |
| .0099305 | .969 | .752 |
| .0100370 | .969 | .750 |
| .0101610 | .969 | .748 |
| .0103078 | .969 | .745 |
| .0103470 | .969 | .743 |
| .0103769 | .938 | .743 |
| .0104393 | .938 | .740 |
| .0106732 | .906 | .740 |
| .0109671 | .906 | .738 |
| .0111091 | .906 | .735 |
| .0111564 | .906 | .733 |
| .0112288 | .906 | .731 |
| .0112986 | .906 | .728 |
| .0115024 | .906 | .726 |
| .0117794 | .906 | .723 |
| .0118581 | .906 | .721 |
| .0119005 | .906 | .718 |
| .0119829 | .906 | .716 |
| .0120868 | .906 | .714 |
| .0122239 | .906 | .711 |
| .0123083 | .906 | .709 |
| .0123608 | .906 | .706 |
| .0125186 | .906 | .704 |
| .0127082 | .906 | .701 |
| .0128180 | .906 | .699 |
| .0128979 | .906 | .697 |
| .0129841 | .906 | .694 |
| .0130274 | .906 | .692 |
| .0130649 | .906 | .689 |
| .0131372 | .906 | .687 |
| .0131809 | .906 | .684 |
| .0132219 | .906 | .682 |
| .0132630 | .906 | .680 |
| .0132782 | .906 | .677 |
| .0133610 | .906 | .675 |
| .0136494 | .906 | .672 |
| .0139474 | .906 | .670 |
| .0140678 | .906 | .667 |
| .0141211 | .906 | .665 |
| .0142189 | .906 | .663 |
| .0143930 | .906 | .660 |
| .0145238 | .906 | .658 |
| .0145688 | .906 | .655 |
| .0147122 | .906 | .653 |
| .0148813 | .875 | .650 |
| .0149205 | .875 | .648 |
| .0149772 | .875 | .646 |
| .0150667 | .875 | .643 |
| .0151569 | .875 | .641 |
| .0152142 | .875 | .636 |
| .0152544 | .875 | .633 |
| .0153387 | .875 | .631 |
| .0153932 | .875 | .629 |
| .0154002 | .875 | .626 |
| .0154409 | .875 | .621 |
| .0155745 | .875 | .619 |
| .0156745 | .875 | .617 |
| .0157793 | .875 | .614 |
| .0160219 | .875 | .612 |
| .0161817 | .875 | .609 |
| .0164508 | .875 | .607 |
| .0168051 | .875 | .604 |
| .0170063 | .875 | .602 |
| .0173799 | .875 | .600 |
| .0177578 | .875 | .597 |
| .0180742 | .875 | .595 |
| .0183058 | .875 | .592 |
| .0183666 | .844 | .590 |
| .0186407 | .844 | .587 |
| .0190009 | .844 | .585 |
| .0192321 | .844 | .583 |
| .0193463 | .844 | .580 |
| .0194841 | .844 | .578 |
| .0197825 | .844 | .575 |
| .0200180 | .844 | .573 |
| .0202180 | .844 | .570 |
| .0205461 | .844 | .566 |
| .0207904 | .844 | .563 |
| .0209644 | .844 | .561 |
| .0211538 | .844 | .558 |
| .0212285 | .844 | .556 |
| .0213037 | .844 | .553 |
| .0214352 | .844 | .551 |
| .0215675 | .844 | .549 |
| .0216486 | .844 | .546 |
| .0217054 | .844 | .541 |
| .0219700 | .844 | .539 |
| .0223044 | .844 | .534 |
| .0227053 | .844 | .532 |
| .0232192 | .844 | .529 |
| .0235050 | .844 | .527 |
| .0239392 | .844 | .524 |
| .0246362 | .844 | .522 |
| .0250917 | .844 | .519 |
| .0252887 | .844 | .517 |
| .0255110 | .844 | .515 |
| .0259973 | .844 | .512 |
| .0264024 | .844 | .510 |
| .0266523 | .844 | .507 |
| .0272378 | .844 | .505 |
| .0279100 | .844 | .502 |
| .0283261 | .844 | .498 |
| .0285930 | .844 | .495 |
| .0287941 | .844 | .493 |
| .0290395 | .844 | .490 |
| .0292798 | .844 | .488 |
| .0293758 | .844 | .485 |
| .0294024 | .844 | .483 |
| .0294923 | .844 | .481 |
| .0296460 | .844 | .478 |
| .0298206 | .844 | .476 |
| .0300167 | .844 | .473 |
| .0301286 | .844 | .468 |
| .0303064 | .844 | .466 |
| .0305568 | .844 | .464 |
| .0309257 | .844 | .461 |
| .0321087 | .844 | .459 |
| .0330095 | .844 | .456 |
| .0330952 | .844 | .454 |
| .0332820 | .844 | .449 |
| .0334849 | .844 | .447 |
| .0335942 | .844 | .444 |
| .0336889 | .844 | .442 |
| .0337989 | .844 | .439 |
| .0339323 | .844 | .437 |
| .0341544 | .844 | .434 |
| .0344516 | .844 | .432 |
| .0349636 | .844 | .430 |
| .0354968 | .844 | .427 |
| .0358126 | .844 | .425 |
| .0359454 | .844 | .422 |
| .0360464 | .844 | .420 |
| .0361395 | .844 | .417 |
| .0363679 | .844 | .413 |
| .0368111 | .844 | .410 |
| .0370423 | .844 | .408 |
| .0371798 | .844 | .405 |
| .0377402 | .844 | .403 |
| .0383130 | .844 | .400 |
| .0387975 | .844 | .398 |
| .0392326 | .844 | .396 |
| .0393424 | .844 | .393 |
| .0397107 | .844 | .391 |
| .0401916 | .844 | .388 |
| .0405392 | .844 | .386 |
| .0409073 | .844 | .383 |
| .0411637 | .844 | .381 |
| .0412878 | .844 | .376 |
| .0414125 | .844 | .374 |
| .0415559 | .844 | .371 |
| .0418263 | .844 | .369 |
| .0420690 | .844 | .367 |
| .0424522 | .844 | .364 |
| .0428356 | .844 | .362 |
| .0428547 | .844 | .359 |
| .0429648 | .844 | .357 |
| .0433355 | .844 | .354 |
| .0437080 | .844 | .352 |
| .0449085 | .844 | .350 |
| .0465546 | .844 | .347 |
| .0472332 | .844 | .345 |
| .0475488 | .844 | .342 |
| .0479886 | .844 | .340 |
| .0487023 | .844 | .337 |
| .0497998 | .844 | .335 |
| .0511678 | .844 | .333 |
| .0524991 | .844 | .330 |
| .0531248 | .844 | .328 |
| .0533989 | .844 | .325 |
| .0540507 | .844 | .323 |
| .0546263 | .844 | .320 |
| .0550915 | .813 | .320 |
| .0558427 | .813 | .318 |
| .0570039 | .813 | .316 |
| .0578634 | .813 | .313 |
| .0580731 | .813 | .311 |
| .0582709 | .813 | .308 |
| .0585655 | .813 | .303 |
| .0589002 | .813 | .301 |
| .0592887 | .813 | .299 |
| .0596794 | .813 | .296 |
| .0605443 | .813 | .294 |
| .0612330 | .813 | .291 |
| .0636674 | .813 | .289 |
| .0661459 | .813 | .286 |
| .0669158 | .813 | .284 |
| .0682275 | .813 | .282 |
| .0688419 | .813 | .279 |
| .0690740 | .813 | .277 |
| .0693061 | .813 | .274 |
| .0695093 | .813 | .272 |
| .0699171 | .813 | .269 |
| .0705796 | .781 | .269 |
| .0711842 | .781 | .267 |
| .0713612 | .781 | .265 |
| .0715700 | .781 | .262 |
| .0728405 | .750 | .262 |
| .0743986 | .750 | .260 |
| .0754889 | .750 | .257 |
| .0764930 | .750 | .252 |
| .0771779 | .750 | .250 |
| .0778526 | .750 | .248 |
| .0782706 | .750 | .245 |
| .0789731 | .750 | .243 |
| .0801571 | .750 | .240 |
| .0820932 | .750 | .235 |
| .0837195 | .750 | .233 |
| .0841852 | .750 | .231 |
| .0851620 | .750 | .228 |
| .0863561 | .750 | .226 |
| .0871580 | .750 | .223 |
| .0881654 | .750 | .221 |
| .0907014 | .719 | .221 |
| .0931443 | .719 | .214 |
| .0939225 | .719 | .211 |
| .0941306 | .719 | .209 |
| .0946693 | .719 | .206 |
| .0954393 | .719 | .204 |
| .0960038 | .719 | .201 |
| .0966117 | .719 | .199 |
| .0977172 | .719 | .197 |
| .0985488 | .719 | .194 |
| .0990682 | .719 | .192 |
| .0995878 | .688 | .192 |
| .0998630 | .688 | .189 |
| .1004930 | .688 | .187 |
| .1025398 | .688 | .184 |
| .1051199 | .688 | .182 |
| .1066054 | .688 | .180 |
| .1072042 | .688 | .177 |
| .1075057 | .688 | .175 |
| .1080880 | .688 | .172 |
| .1097100 | .688 | .170 |
| .1113170 | .688 | .167 |
| .1118941 | .688 | .165 |
| .1128593 | .688 | .163 |
| .1167274 | .688 | .160 |
| .1202026 | .688 | .158 |
| .1208692 | .688 | .155 |
| .1236622 | .688 | .153 |
| .1267934 | .688 | .150 |
| .1276305 | .688 | .148 |
| .1282548 | .688 | .146 |
| .1301874 | .688 | .143 |
| .1316910 | .688 | .141 |
| .1324128 | .688 | .138 |
| .1331085 | .688 | .136 |
| .1338641 | .688 | .133 |
| .1356993 | .688 | .131 |
| .1379261 | .688 | .129 |
| .1401277 | .688 | .126 |
| .1439469 | .688 | .124 |
| .1467454 | .688 | .121 |
| .1486940 | .688 | .119 |
| .1506251 | .688 | .117 |
| .1511582 | .688 | .114 |
| .1519964 | .688 | .112 |
| .1524016 | .688 | .109 |
| .1535666 | .656 | .109 |
| .1569036 | .625 | .109 |
| .1594334 | .594 | .109 |
| .1598548 | .594 | .107 |
| .1603405 | .594 | .104 |
| .1615206 | .594 | .102 |
| .1623723 | .594 | .100 |
| .1632306 | .594 | .097 |
| .1684273 | .594 | .095 |
| .1732791 | .594 | .092 |
| .1737629 | .594 | .087 |
| .1743158 | .594 | .085 |
| .1756104 | .594 | .083 |
| .1777982 | .563 | .083 |
| .1792450 | .563 | .080 |
| .1797083 | .563 | .078 |
| .1811041 | .531 | .078 |
| .1844703 | .531 | .075 |
| .1877573 | .531 | .073 |
| .1920223 | .531 | .070 |
| .1979688 | .531 | .068 |
| .2015497 | .531 | .066 |
| .2040551 | .531 | .063 |
| .2082553 | .531 | .061 |
| .2178024 | .531 | .058 |
| .2280449 | .500 | .058 |
| .2354068 | .469 | .058 |
| .2428342 | .469 | .056 |
| .2469013 | .469 | .053 |
| .2509870 | .469 | .051 |
| .2556251 | .438 | .051 |
| .2579804 | .406 | .051 |
| .2603945 | .375 | .051 |
| .2653267 | .375 | .049 |
| .2691398 | .375 | .046 |
| .2731955 | .375 | .044 |
| .2781062 | .375 | .041 |
| .2797330 | .375 | .039 |
| .2830118 | .375 | .036 |
| .2861571 | .375 | .034 |
| .2867517 | .375 | .032 |
| .2874431 | .375 | .029 |
| .2926413 | .375 | .027 |
| .3024513 | .375 | .024 |
| .3078062 | .344 | .024 |
| .3091985 | .344 | .022 |
| .3124672 | .313 | .022 |
| .3158013 | .313 | .019 |
| .3212719 | .313 | .017 |
| .3261774 | .281 | .017 |
| .3317969 | .250 | .017 |
| .3495355 | .250 | .015 |
| .3660407 | .250 | .012 |
| .3762724 | .219 | .012 |
| .3866936 | .188 | .012 |
| .3941734 | .156 | .012 |
| .4062662 | .156 | .010 |
| .4169540 | .125 | .010 |
| .4308056 | .094 | .007 |
| .4493641 | .063 | .007 |
| .4602677 | .031 | .007 |
| .4696520 | .031 | .005 |
| .4876950 | .031 | .002 |
| .5023009 | .000 | .002 |
| 1.0000000 | .000 | .000 |
| The test result variable(s): Predicted probability has at least one tie between the positive actual state group and the negative actual state group. | | |
| a. The smallest cutoff value is the minimum observed test value minus 1, and the largest cutoff value is the maximum observed test value plus 1. All the other cutoff values are the averages of two consecutive ordered observed test values. | | |
