# Supplementary material for: Inflammatory and nutritional indices as predictors of poor prognosis in pyogenic liver abscesses: a retrospective cohort study
Source: PeerJ. 2026 Oct 27;14:e21852. doi: 10.7717/peerj.21852 (PMC13721881; doi:10.7717/peerj.21852)
Supplement: Supplementary file 6 [file peerj-14-21852-s006.docx]

**Supplementary Table 3. Model performance metrics**

| Metric | Value |
| --- | --- |
| Sample size (N) | 444 |
| Event rate (%) | 7.21 (32/444) |
| Apparent AUC | 0.822 (95% CI: 0.735–0.909) |
| Optimism-corrected AUC | 0.817 |
| Mean optimism (1,000 bootstraps) | 0.0056 |
| Brier score | 0.0548 |
| Hosmer-Lemeshow χ² (df = 8) | 13.652 |
| Hosmer-Lemeshow p-value | 0.0913 |
| Decision curve analysis | Net benefit > 0 across thresholds, including at cutoff 0.188 |
